# ERK MAP Kinase Signaling Regulates RAR Signaling to Confer Retinoid Resistance on Breast Cancer Cells

**DOI:** 10.3390/cancers14235890

**Published:** 2022-11-29

**Authors:** Akira Hirota, Jean-Emmanuel Clément, Satoshi Tanikawa, Takayuki Nonoyama, Tamiki Komatsuzaki, Jian Ping Gong, Shinya Tanaka, Masamichi Imajo

**Affiliations:** 1 Institute for Chemical Reaction Design and Discovery (WPI-ICReDD), Hokkaido University, Sapporo 001-0021, Japan; 2 Research Center of Mathematics for Social Creativity, Research Institute for Electronic Science, Hokkaido University, Sapporo 001-0020, Japan; 3 Faculty of Advanced Life Science, Hokkaido University, Sapporo 001-0021, Japan; 4 Department of Cancer Pathology, Faculty of Medicine, Hokkaido University, Sapporo 060-8638, Japan

**Keywords:** breast cancer, retinoids, retinoic acid receptor (RAR), ERK MAP kinase, breast cancer subtypes

## Abstract

**Simple Summary:**

Breast cancer is among the most common cancers and the leading cause of cancer-related death in women worldwide. Among potential anticancer drugs considered promising in breast cancer treatment are retinoids that act mainly through nuclear retinoic acid receptors (RARs). Clinical trials, however, showed that cancer cells often acquire resistance to retinoid therapy. Therefore, elucidation of underlying mechanisms of retinoid resistance is needed to develop more effective use of retinoids in cancer treatment. In this study, we identify activation of ERK MAP kinase signaling as a novel mechanism for retinoid resistance of breast cancer cells. We show that ERK signaling regulates RAR signaling and inhibition of ERK potentiates tumor-suppressive functions of RARs in breast cancer cells. Moreover, we also reveal that suppression of RAR signaling coincides with activation of ERK signaling in specific subtypes of breast cancers and that these changes are associated with poor prognoses of breast cancer patients.

**Abstract:**

Retinoic acid (RA) and its synthetic derivatives, retinoids, have been established as promising anticancer agents based on their ability to regulate cell proliferation and survival. Clinical trials, however, have revealed that cancer cells often acquire resistance to retinoid therapy. Therefore, elucidation of underlying mechanisms of retinoid resistance has been considered key to developing more effective use of retinoids in cancer treatment. In this study, we show that constitutive activation of ERK MAP kinase signaling, which is often caused by oncogenic mutations in *RAS* or *RAF* genes, suppresses RA receptor (RAR) signaling in breast cancer cells. We show that activation of the ERK pathway suppresses, whereas its inhibition promotes, RA-induced transcriptional activation of RAR and the resultant upregulation of RAR-target genes in breast cancer cells. Importantly, ERK inhibition potentiates the tumor-suppressive activity of RA in breast cancer cells. Moreover, we also reveal that suppression of RAR signaling and activation of ERK signaling are associated with poor prognoses in breast cancer patients and represent hallmarks of specific subtypes of breast cancers, such as basal-like, HER2-enriched and luminal B. These results indicate that ERK-dependent suppression of RAR activity underlies retinoid resistance and is associated with cancer subtypes and patient prognosis in breast cancers.

## 1. Introduction

Breast cancer is the leading cause of cancer-related death in women with an estimated 2.3 million new cases worldwide (representing 11.7% of all cancer cases) in 2020 [1]. Breast cancer has been classified into five subtypes according to molecular signatures: luminal A/B, basal-like (triple-negative), human epidermal growth factor receptor 2 (HER2)-enriched, and normal-like [2,3,4]. In general, the basal-like and HER2-enriched subtypes have a poor prognosis with frequent cancer recurrence, whereas normal-like and luminal subtypes have a more favorable prognosis. Among luminal subtypes, the luminal B subtype has been associated with poorer prognosis than the luminal A subtype. Reflecting the distinct mechanisms of carcinogenesis, individual subtypes of breast cancers exhibit different sensitivity to therapeutic intervention, including anti-hormone therapy, molecularly targeted therapy, and chemotherapy [4]. For instance, the HER2-enriched subtype often shows dependency on HER2 signaling activity, and therefore HER2 kinase inhibitors and anti-HER2 antibodies have been used to treat this type of breast cancer [4]. Luminal A and B subtypes are positive for estrogen receptor (ER) expression and often sensitive to hormone therapy [4]. The basal-like subtype expresses neither HER2 nor ER, and thus is resistant to both hormone therapy and HER2-targeted therapy, which has been causative for poor prognosis of this type of breast cancer. Thus, induction of phenotype switching from the basal subtype into more favorable subtypes can be a promising strategy for breast cancer treatment [5]. Elucidation of genetic programs that operate in each subtype has been considered key to developing novel strategies for breast cancer treatment and to improving patient outcomes.

Among factors that might regulate breast carcinogenesis is retinoic acid receptor (RAR)/retinoid X receptor (RXR) signaling. RARs and RXRs constitute the subfamilies of nuclear receptors, whose activity is regulated by the binding of retinoids (an active metabolite of vitamin A, retinoic acid (RA), and its synthetic derivatives) [6,7]. In the absence of retinoids, the RAR/RXR heterodimer recruits transcriptional corepressors to the target promoters [6,7]. After ligand binding, conformational changes in the ligand binding domain of RARs induce dislodgement of corepressors and binding of coactivators, promoting expression of target genes [6,7]. While triggering transcription of target genes, ligand binding also induces degradation of RARs and RXRs, which serves as negative feedback mechanisms to control RAR signaling [8]. RAR/RXR signaling has been shown to play an important role in the embryonic development and homeostasis of many tissues during postnatal life [6,7]. Importantly, activation of RAR/RXR signaling triggered by retinoids often exerts tumor-suppressive effects in mice, and loss of normal RAR function has been implicated in the progression of a diverse range of human cancers, including breast, lung, and colorectal carcinomas [9,10]. Retinoids also show potent anti-proliferative and pro-apoptotic activity on breast cancer cells [11,12]. In line with this, several potential tumor-suppressive genes including *HOXA5* and *ELF3* have been shown to be upregulated by retinoids in breast cancer cells [13,14]. However, clinical studies of retinoids in the treatment of breast cancers were mostly disappointing, likely because of the acquisition of resistance to retinoids during carcinogenesis and/or treatment [10,15]. Therefore, there is an urgent need for elucidation of underlying mechanisms of the retinoid resistance in breast cancer cells. In addition, identification of molecular markers that predict sensitivity to retinoids would be valuable for successful use of retinoids in cancer treatment [16].

In pursuit of molecular mechanisms of retinoid resistance in cancer cells, we have recently shown that ERK MAP kinase signaling and RAR signaling mutually suppress each other via transcriptional induction of their negative regulators [17]. Notably, this mutual antagonism between ERK and RAR signaling regulates colorectal cancer (CRC) cell fates [17]. Acting downstream of growth factor receptor/RAS/RAF/MEK signaling, ERK MAP kinases play a critical role in cell proliferation and survival [18,19], and are often activated by oncogenic mutations in *RAS* or *RAF* genes in cancer cells [19,20,21]. We found that ligand-dependent activation of RAR induces expression of a dual-specificity phosphatase, MAP kinase phosphatase 4, thereby inducing dephosphorylation and inactivation of ERK [17]. Conversely, strong activation of ERK suppresses ligand-dependent activation of RARs in a histone deacetylase (HDAC)- and nuclear receptor-interacting protein 1 (NRIP1)-dependent manner [17]. Importantly, RAR activation promotes, whereas ERK activation suppresses, differentiation of CRC cells, suggesting a role of antagonistic interactions between ERK and RAR signaling in CRC cell fate decisions [17]. In this study, we aimed to extend the relationships between RAR and ERK signaling to breast cancers and reveal their pathological relevance. We show that ERK activation caused by oncogenic mutants of RAS, RAF, and MEK inhibits RAR signaling in breast cancer cells. By contrast, inhibition of ERK augments the RA-dependent induction of RAR-target genes and tumor-suppressive activity of RA. Moreover, our analyses suggest that ERK activation and suppression of RAR are hallmarks of specific breast cancer subtypes and are associated with patient prognosis.

## 2. Materials and Methods

### 2.1. Cell Culture

All cell lines used in this study (MCF-7, MDA-MB-231, MCF10A, and HEK293T) were obtained from American Type Culture Collection. Cells were cultured in Dulbecco’s modified Eagle medium (DMEM) supplemented with 10% fetal bovine serum, 2 mM glutamine, and antibiotics (100 U/mL of penicillin).

### 2.2. Plasmids, Antibodies and Reagents

pGL3-basic-RARE3 reporter plasmid and expression plasmids for constitutively active MEK (LA-SDSE), wild-type RARα and RXRα, and a dominant negative form of RARα have been described previously [17,22]. Antibodies specific for ERK1/2 (Cell Signaling Technology, Danvers, MA, USA), phospho ERK1/2 (Cell Signaling Technology), HA (Santa Cruz Biotechnology, Dallas, TX, USA) and α-tubulin (Sigma-Aldrich, St. Louis, MO, USA) were used for immunoblotting. For immunoblotting data, uncropped images are shown in Figure S1. PD0325901 and all-trans retinoic acid were purchased from Stemgent and Sigma-Aldrich, respectively. The drugs were stocked in DMSO.

### 2.3. RT-PCR Analysis

Total RNA was extracted by using an RNeasy mini kit (Qiagen, Hilden, Germany). cDNA was synthesized by using High-Capacity cDNA Reverse Transcription Kit (Thermo Fisher Scientific, Waltham, MA, USA). Prepared cDNA was then analyzed by quantitative PCR by using Step One Plus real time PCR system (Thermo Fisher Scientific) with SYBR Green PCR Kit (Applied Biosystems, Waltham, MA, USA). Expression values obtained were normalized to that of *RPS26*. Primer sequences are available from the authors on request.

### 2.4. Luciferase Assay

Cells were transfected with a reporter plasmid that expresses firefly luciferases under the control of retinoic acid responsive elements (RAREs) (pGL3-basic-RARE3) [17] and a control plasmid that expresses Renilla luciferases under the constitutive promoter (pRL-TK) by using FuGENE HD (Promega, Madison, WI, USA). Firefly and Renilla luciferase activity was measured by using the Dual-Glo luciferase assay system (Promega). Firefly luciferase activity was normalized to the co-expressed Renilla luciferase activity.

### 2.5. Synthesis of Poly(DMAAm-co-APTMA) Hydrogels

The hydrogels with varied strength of positive charges were synthesized by free-radical copolymerization from aqueous solutions containing neutral monomer N,N′-dimethylacrylamide (DMAAm), cationic monomer (3-acrylamidopropyl) trimethylammonium (APTMA), crosslinker N,N′-methylenebisacrylamide (MBAA) (4 mol%), initiator ammonium persulfate (APS) (0.4% (*w*/*v*)) and N,N,N′,N′-tetramethylethylenediamine (TEMED) (0.1% (*v*/*v*)). The total concentration of monomers (DMAAm and APTMA) was fixed at 1 M, and the molar ratio of the two monomers was varied. The molar percentages (mol%) are in relative to the monomer concentration. Sheet-shaped hydrogels were synthesized in the reaction cells that were assembled by two parallel glass plates and separated by a spacer of 1 mm thickness. In this paper, the hydrogels are named according to the ratio of monomers (e.g., if the ratio of cationic APTMA to neutral DMAAm is 1:9, the gel is labeled as C1N9 gel). After gelation, the synthesized hydrogels were extensively washed with phosphate-buffered saline (PBS) to remove unpolymerized monomers. Gel disks were punched out from the gel sheet, sterilized by autoclaving (120 °C, 20 min), placed in polystyrene tissue culture dishes, and used for cell culture.

### 2.6. RNA-Sequencing Analysis

For RNA-sequencing (RNA-seq), MCF-7 cells were cultured on conventional polystyrene (PS) cell culture dishes or APTMA-DMAAm (C5N5) hydrogels for three days. Total RNA was extracted by using the NucleoSpin RNA Plus kit (Takara Bio, Kusatsu, Japan) according to manufacturer’s instructions. The library construction and RNA-sequencing were performed at Beijing Genomics Institute (BGI). After library construction, 150-bp paired-end reads were sequenced using BGISEQ-T7 platform. After sequencing, the adaptor sequences and raw reads were filtered to get clean reads. The total number of clean reads was about 40 megabases for one sample. The clean reads were mapped to the reference human genome (GCF_000001405.39_GRCh38.p13) with HISAT [23] and then to the reference genes with Bowtie2 [24]. Differential expression was determined with DESeq2 [25]. We identified genes whose expression levels were up- or downregulated by more than 2-fold with statistical significance (*p* < 0.05) as hydrogel-upregulated or -downregulated genes. Heatmaps of differentially expressed genes (DEGs) were generated using the R statistical software. Gene set enrichment analysis (GSEA) [26] was done by sorting the output from DESeq2. To analyze the relationships between patient prognoses and expression status of genes upregulated by hydrogel, gene expression datasets of breast cancer patients were obtained from The Cancer Genome Atlas (TCGA) websites [27,28]. We identified hydrogel-induced genes whose expression levels were upregulated in breast cancer patients (average *Z* scores > 0). We then classified breast cancer patients based on the average *Z* scores of these genes into two categories (*Z* > or <0). Kaplan–Meier survival analysis of classified patients was performed using the R software. For statistical analysis, the survival curves were compared using the log-rank test.

### 2.7. Analysis of Human Breast Cancer Microarray and RNA-Sequencing Datasets

Microarray datasets of human breast cancers were obtained from the Gene Expression Omnibus (GEO) (GSE3744 and GSE1456) and analyzed by GeneSpring GX 10 (Agilent Technologies, Santa Clara, CA, USA) software. Gene expression data of cancer samples were normalized to the median of normal control tissues (GSE3744) or to the median of all samples (GSE1456). For the calculation of expression signals of all genes, the GCRMA (log2) algorithm was used. Prior to analysis, probe sets with signals present at background noise levels were filtered out. The list of RA-dependent upregulated genes in MCF-7 breast cancer cells have been previously reported [29]. ERK-dependent upregulated genes in mammary epithelial cells were identified by using the published microarray datasets (GSE12764). We identified those genes whose expression levels in MCF10A cells expressing constitutively active MEK were upregulated by more than 3-folds with statistical significance (one way ANOVA, BH-FDR = 0.05), compared to those in control cells, as ERK-dependent upregulated genes in mammary epithelial cells. For analyses in Figure 6C–G, we used the lists of the RA- and ERK-dependent upregulated genes, whose expression levels in breast cancers were decreased and increased, respectively, by more than 1.5-fold compared to those in normal mammary epithelia (Figure 6B), to examine the relationship between the changes in RAR and ERK signaling and clinical information. For Kaplan–Meier survival analysis, we classified breast cancer samples into four groups based on expression profiles of the RA- and ERK-dependent upregulated genes. We counted the RA-dependent upregulated genes whose expression values are −0.5 or under (log2) in each sample, and examined whether the number of these genes in each sample was significantly higher compared to the average number in all samples by Fisher’s exact test (*p* < 0.1). Samples with the significantly increased numbers of these genes were defined as RA-D (decreased), and other samples were defied as RA-ND (not decreased). Similarly, samples with the significantly increased numbers of the ERK-dependent upregulated genes, whose expression values are 1 or over, were defined as ERK-I (increased), and other samples were defined as ERK-NI (not increased). Thus, we classified breast cancer samples into four categories: RA-ND/ERK-NI, RA-ND/ERK-I, RA-D/ERK-NI, and RA-D/ERK-I. The log-rank test was used to examine statistically significant differences among the survival curves. For the analysis of larger scale gene expression datasets, gene expression datasets of more than 1500 human breast cancer patients were downloaded from TCGA [27,28]. The statistical analysis was performed in R software version 4.2.1. Heatmaps and a correlation matrix of the RA- and ERK-dependent upregulated genes, whose expression levels in breast cancers were decreased and increased, respectively, were generated by R packages, complexHeatmap and pheatmap. Uniform Manifold Approximation and Projection (UMAP) of breast cancer patients based on the expression levels of the RA- and ERK-dependent upregulated genes was performed by using the umap package in R with the default parameters of the algorithm. To examine the relationship between prognoses of cancer patients and the expression status of the RA- and ERK-upregulated genes, we performed a cluster analysis by k-means algorithm on the full set of patients. The choice of cluster number is based on a cluster-wise assessment of cluster stability with clusterboot function from the fpc R package. Up to 3 clusters, the mean bootstrap Jaccard index were higher than 0.85 for all individual clusters [30,31]. From 4 to 10 clusters, some individual clusters show a mean bootstrap Jaccard index inferior or equal to 0.5, implying some instabilities or uncertainty in the clustering [30,31]. The number of clusters to assess the relation between RA- and ERK-upregulated genes was then set to 3. The Krukal-Wallis test was used to detect the presence of central difference between the 3 mentioned groups for survival and relapse free survival time, among uncensored observations. If the *p* value of Krukal-Wallis test was below 0.05, the standard pairwise Wilcoxon rank sum test between all possible pairs of the 3 groups was applied. Kaplan–Meier estimator from survival R package was used to estimate survival and relapse free survival time functions. To assess whether the difference between the 3 survival time functions and the difference between the 3 free-relapse time functions are statistically significant, a log rank test was used.

### 2.8. Xenograft Mouse Model of Breast Cancers

Female BALB/c-*nu/nu* mice (8 weeks old) were subjected to subcutaneous transplantation of MDA-MB-231 cells. Cells were cultured under the standard culture condition and treated with vehicle (DMSO), RA (1 μM), and/or PD0325901 (0.3 μM) for 9 days. After detachment by trypsinization, cells were first suspended in PBS, and then mixed in a 1:1 ratio with Matrigel. A 0.1 mL cell suspension containing 2 × 10^5^ cells was subcutaneously injected into the flank of mice. The tumors were allowed to grow for up to 16 weeks. After euthanasia of mice, the developed tumors were excised and weighed. This study was approved by the institutional animal care and use committee of Hokkaido University.

## 3. Results

### 3.1. ERK Signaling Regulates RAR-Target Gene Expression in Breast Cancer Cells

**Figure 1:**
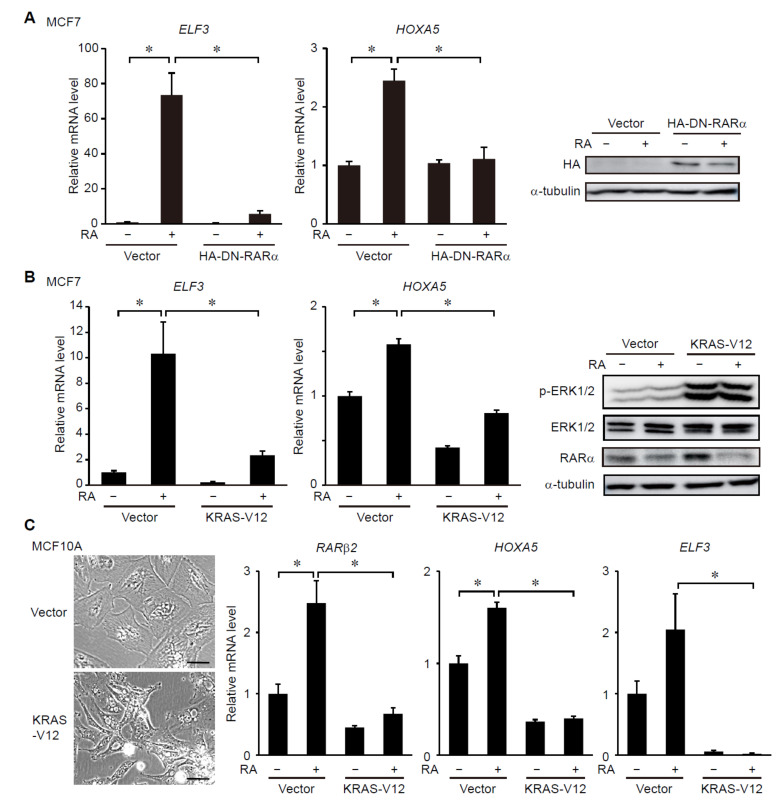
Ligand-dependent induction of RAR-target genes is suppressed by activation of RAS-ERK signaling. (**A**) A HA-tagged dominant negative form of RARα (HA-DN-RARα) was expressed in MCF7 cells by using lentivirus. Cells were treated with DMSO or RA (1 μM) for 24 h prior to the analyses. Total RNA and proteins were analyzed by RT-PCR (left) and immunoblotting (right), respectively. *, *p* < 0.05 (one-way ANOVA followed by Tukey post hoc analyses) (mean + s.e.m., *n* = 4). (**B**) KRAS-V12 was expressed in MCF7 cells by using lentivirus. 4 days after infection of lentivirus, cells were treated with DMSO or RA (1 μM) for 24 h. Total RNA and proteins were analyzed by RT-PCR (left) and immunoblotting (right), respectively. *, *p* < 0.05 (one-way ANOVA followed by Tukey post hoc analyses) (mean + s.e.m., *n* = 4). (**C**) KRAS-V12 was expressed in MCF10A cells by using lentivirus. 3 days after infection of lentivirus, cells were treated with DMSO or RA (1 μM) for 24 h. The indicated mRNA levels were analyzed by RT-PCR. (left) Morphology of MCF10A cells expressing KRAS-V12 or control cells. Scale bars: 50 μm. *, *p* < 0.05 (one-way ANOVA followed by Tukey post hoc analyses) (mean + s.e.m., *n* = 3). Uncropped immunoblot images are shown in Supplementary Material (Figure S1).

To examine the relationship between RAR and ERK signaling in breast cancer cells, we first verified that expression of potential tumor-suppressive genes, *HOXA5* and *ELF3*, was induced by RA treatment in an RAR-dependent manner in MCF7 breast cancer cells. To examine dependency on RAR, a dominant negative form of RARα (DN-RARα), which lacks the C-terminus ligand-binding domain [17], was used. The DN-RARα competes with endogenous RARs for binding to the RA responsive element (RARE) in the target promoters without transactivating transcription [17]. We found that expression of *HOXA5* and *ELF3* was promoted by treatment with RA in MCF7 cells, whereas expression of DN-RARα suppresses the RA-dependent induction of these genes (Figure 1A). This suggests that transcriptional activation of RARs mediates RA-induced expression of *HOXA5* and *ELF3*.

**Figure 2:**
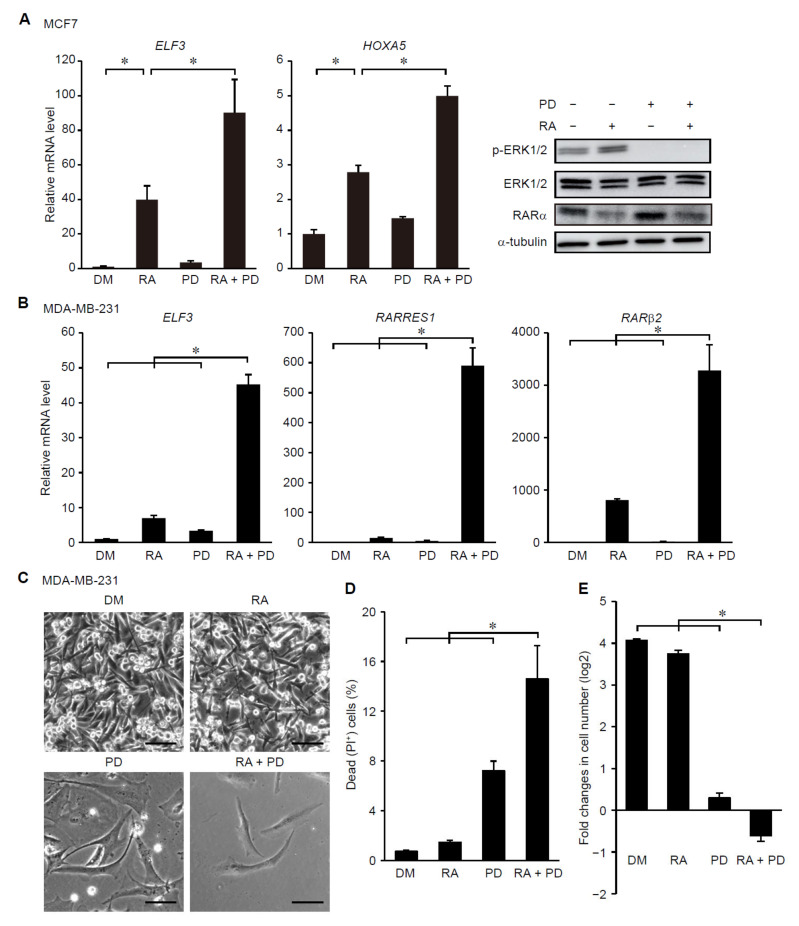
Inhibition of the ERK pathway potentiates RA-induced expression of RAR-target genes, growth suppression and cell death in breast cancer cells. (**A**) MCF7 cells were treated with RA (1 μM), a MEK inhibitor (PD0325901, 3 μM), and/or DMSO for 24 h. Total RNA and cell lysates were prepared and analyzed by RT-PCR (left) and immunoblotting (right), respectively. *, *p* < 0.05 (one-way ANOVA followed by Tukey post hoc analyses) (mean + s.e.m., *n* = 4). Uncropped immunoblot images are shown in Supplementary Material (Figure S1). (**B**–**E**) MDA-MB-231 cells were treated with RA (1 μM), a MEK inhibitor (PD0325901, 3 μM), and/or DMSO for 8 days (**B**,**C**,**E**) or 6 days (**D**). Total RNA was prepared and analyzed by RT-PCR (B). (**C**) Morphology of MDA-MB-231 cells treated with or without RA and a MEK inhibitor. Scale bars: 50 μm. (**D**) Survival rates of MDA-MB-231 cells treated with or without RA and/or a MEK inhibitor. (**E**) Fold changes in cell number after 8 days culture in the absence or presence of RA and/or a MEK inhibitor. *, *p* < 0.05 (one-way ANOVA followed by Tukey post hoc analyses) (mean + s.e.m., *n* = 4 (**B**,**E**); *n* = 6 (**D**)).

We then examined whether constitutive activation of ERK signaling affects RA-induced expression of RAR-target genes in breast cancer cells. To address this, an oncogenic mutant form of KRAS (KRAS-V12) was stably expressed in MCF7 cells, which led to constitutive activation of ERK (Figure 1B). Expression of KRAS-V12 strongly suppressed RA-induced expression of *HOXA5* and *ELF3* (Figure 1B). Importantly, the KRAS-V12 expression did not affect the basal protein level of RARα and its ligand-induced degradation [8] (Figure 1B), suggesting that KRAS signaling regulates the activity, but not the abundance, of RARs. To assess the generality of this result, we examined the effects of ERK activation on RAR-target gene expression in MCF10A normal mammary epithelial cells. The expression of KRAS-V12 changed the morphology of MCF10A cells from cuboidal to an elongated spindle-like shape suggestive of epithelial mesenchymal transition as reported previously [32] (Figure 1C). The KRAS-V12 expression suppressed RA-induced expression of RAR-target genes in MCF10A cells (Figure 1C), demonstrating the generality of KRAS signaling-dependent regulation of RAR signaling in mammary epithelial cells. By contrast, inhibition of ERK signaling by an inhibitor for MEK synergized with RA treatment to promote expression of the RAR-target genes in MCF7 cells (Figure 2A). Inhibition of ERK did not affect the protein level of RARα (Figure 2A), again showing that ERK does not regulate the abundance of RARs. Moreover, we also found that ERK inhibition strongly enhanced the RA-induced expression of RAR-target genes in MDA-MB-231 cells, which are highly aggressive, triple negative breast cancer (TNBC) cells harboring an oncogenic *KRAS* mutation [33] (Figure 2B). Notably, treatment with RA and a MEK inhibitor synergistically induced cell death and proliferation arrest in MDA-MB-231 cells (Figure 2C–E). These results suggest that ERK signaling negatively regulates ligand-dependent induction of RAR-target genes and that inhibition of ERK potentiates the effects of retinoids on breast cancer cells.

### 3.2. ERK Signaling Regulates Transcriptional Activity of RARs

**Figure 3:**
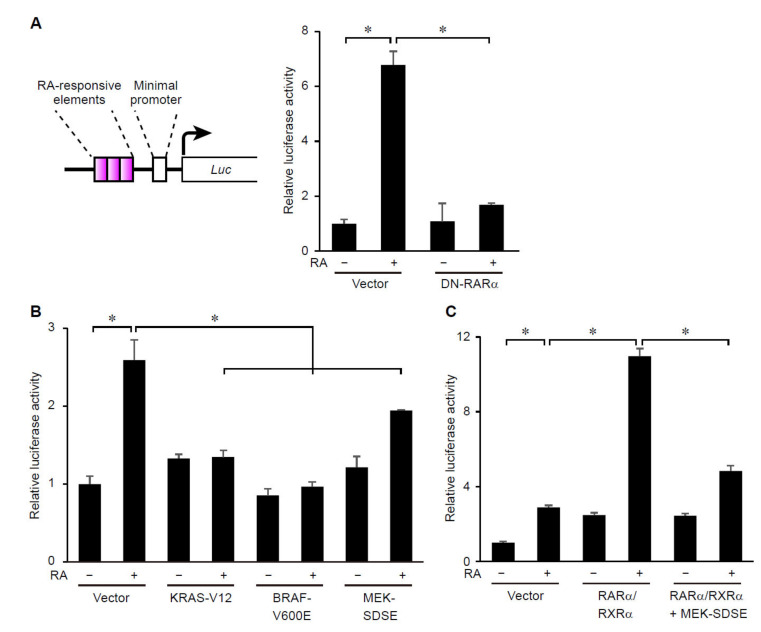
Activation of RAS-ERK signaling suppresses transcriptional activity of RARs. (**A**–**C**) The RARE reporter assays to measure transcriptional activity of RAR (**A**, left) was performed in MCF7 cells. (left) Schematic representation of the RAR reporter construct in which transcription of the luciferase gene (indicated by arrow) was regulated by a minimal promoter and triplicated RAREs (whose locations are indicated by dot lines). Cells were transfected with empty vectors, or expression plasmids for DN-RARα, KRAS-V12, BRAF-V600E, MEK-SDSE, wild type RARα and/or RXRα. Cells were treated with 1 μM of RA for 18 h prior to the measurements. *, *p* < 0.05 (one-way ANOVA followed by Tukey post hoc analyses) (mean + s.e.m., *n* = 3).

We next examined whether ERK signaling affects transcriptional activity of RARs. To this end, RAR transcriptional activity was evaluated by reporter assays with the construct that expresses luciferase under the control of RA responsive elements (RAREs) [17,34]. As expected, treatment with RA increased activity of the reporter expressed by this construct (Figure 3A). Expression of DN-RARα suppressed the RA-dependent increase in the reporter activity in MCF7 cells (Figure 3A), suggesting that the reporter construct could measure endogenous RAR transcriptional activity in our experimental settings. We then examined whether expression of constitutively active forms of upstream regulators of the RAS/RAF/MEK/ERK pathway affects RAR transcriptional activity. The results showed that expression of constitutively active KRAS (KRAS-V12), BRAF (BRAF-V600E), and MEK1 (MEK-SDSE) [22] suppressed ligand-dependent transcriptional activation of RARs (Figure 3B). Importantly, overexpression of wild-type RARα and its heterodimeric partner, RXRα, promoted RAR activity in MCF7 cells (Figure 3C), suggesting that expression levels of endogenous RARα and RXRα are major limiting factors for RAR/RXR-mediated transcription in these cells. Expression of constitutively active MEK suppressed the increase in RAR activity induced by overexpressed RARα and RXRα (Figure 3C). These results suggest that activation of ERK signaling suppresses ligand-induced transcriptional activation of the RAR/RXR heterodimer in breast cancer cells.

### 3.3. Suppression of ERK Signaling Enhances Ligand-Dependent Activation of RAR Signaling in Breast Cancer Cells with Elevated Cancer Stem Cell Properties

**Figure 4:**
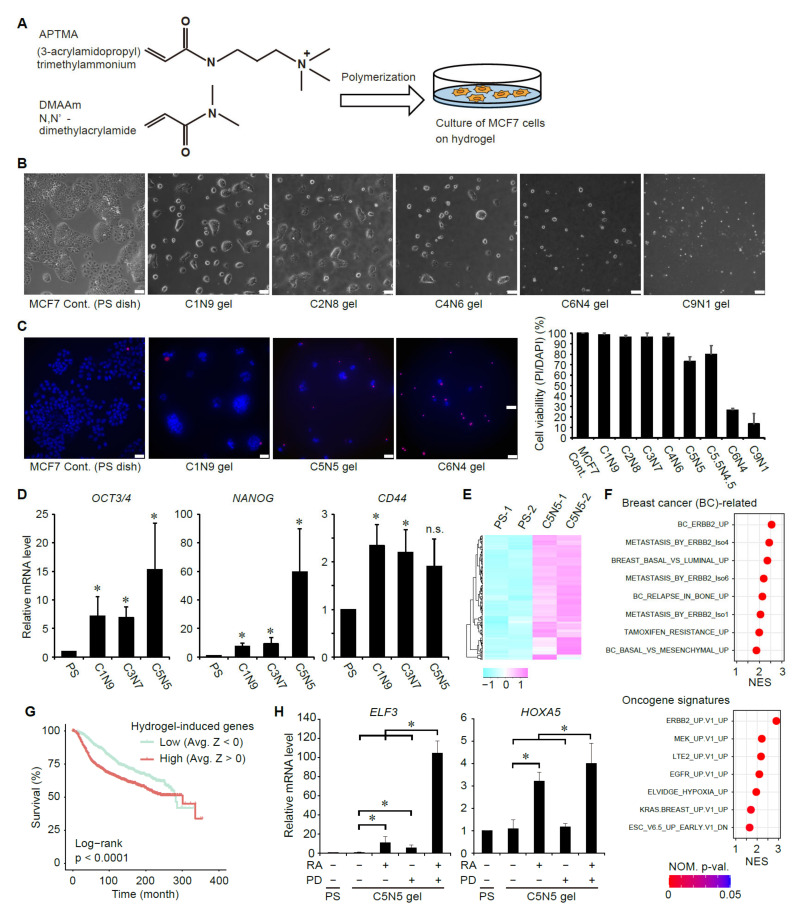
ERK inhibition promotes ligand-dependent RAR-target gene expression in MCF7 cells expressing high levels of CSC markers. (**A**) Schematic representation of the synthesis of the poly(DMAAm-co-APTMA) hydrogel and its use in cell culture. (**B**) Morphology of MCF7 cells cultured on the hydrogels with varying ratios of APTMA to DMAAm. (**C**) Survival rates of MCF7 cells cultured on the poly(DMAAm-co-APTMA) gels. Dead cell nuclei were stained by propidium iodide (PI). (**D**) Expression levels of CSC markers in MCF7 cells cultured on the poly(DMAAm-co-APTMA) gels for 3 days were analyzed by qRT-PCR. *, *p* < 0.05 (one-way ANOVA followed by Tukey post hoc analyses) (mean + s.d., *n* = 3). (**E**) A heatmap showing normalized expression values (*Z*-scores) of genes induced by the C5N5 hydrogel. The data were derived from RNA-sequencing analyses of MCF7 cells cultured on PS dishes or the C5N5 hydrogel. (**F**) Gene sets significantly upregulated in MCF-7 cells cultured on the C5N5 hydrogel. NES: normalized enrichment score. (**G**) Kaplan–Meier survival curves among breast cancer patients are shown. The patient samples are classified in two categories based on expression profiles of the hydrogel-induced genes (high (*Z* > 0), low (*Z* < 0)), as described. *p* value was obtained from the log-rank test. (**H**) Expression levels of the RAR-target genes in MCF7 cells cultured on the hydrogel and treated with or without RA and/or a MEK inhibitor. Cells were cultured on the hydrogels for 3 days and then treated with RA (1 μM), a MEK inhibitor (PD0325901, 3 μM), and/or DMSO for 24 h. *, *p* < 0.05 (one-way ANOVA followed by Tukey post hoc analyses) (mean + s.d., *n* = 3).

Cancer stem cells (CSCs) were first discovered in acute myeloid leukemia [35]. Since then, the CSC theory has been extended to many cancers including breast cancer [36,37,38,39]. CSCs are a small subpopulation of cancer cells with prominent self-renewing and oncogenic potential needed to drive cancer initiation and generate differentiated non-CSC cells [40,41]. CSCs have been considered resistant to many anti-cancer drugs, and thus implicated in cancer relapse [40,41]. To examine the effects of RA and a MEK inhibitor on breast CSCs, we aimed to induce CSC-like phenotypes in MCF7 cells. In breast cancer research, mammosphere formation assay has been used to propagate normal mammary epithelial stem cells and CSCs in vitro [42,43]. Recently, we have shown that culture on certain synthetic hydrogels rapidly induces expression of CSC markers, such as *OCT4* and *NANOG*, more strongly than the conventional sphere culture and confers CSC activity on glioblastoma cells [44]. We thus examined whether culture on hydrogel can induce CSC-like phenotypes in MCF7 cells. To promote cell adhesion to hydrogel, we used positively charged hydrogel composed of a neutral monomer, N,N-dimethylacrylamide (DMAAm), and a cationic monomer, 3-acrylamidopropyl trimethylammonium (APTMA) (Figure 4A). The electric charge of the poly(DMAAm-co-APTMA) gel can be tuned by changing the ratio of two monomers: positive charge of the hydrogel is increased, as the concentration of APTMA increases. We found that MCF7 cells adhere well to the positively charged hydrogels (Figure 4B). However, the proportion of dead cells was increased when the concentration of APTMA exceeds 60% (Figure 4C), suggesting that very strong positive charge would be harmful for cells. We then evaluated the expression levels of CSC markers in cells cultured on the poly(DMAAm-co-APTMA) gels. The expression levels of *OCT4*, *NANOG*, and *CD44* were significantly elevated as positive charge of the hydrogel was increased (Figure 4D), suggesting that culture on the positively charged hydrogel induces CSC markers in MCF7 cells. To comprehensively identify gene expression changes induced by the poly(DMAAm-co-APTMA) hydrogel, we performed gene expression profiling by whole transcriptome RNA sequencing (RNA-seq) technologies. Our RNA-seq analyses identified 154 genes whose expression levels were significantly upregulated by more than 2-fold in MCF7 cells cultured on the hydrogel (Figure 4E, and Table S1). The following gene set enrichment analysis uncovered that the hydrogel culture induces genes related to more malignant, CSC-like phenotypes, including those associated with the breast cancer subtypes with worse prognosis (the ERBB2 and basal subtypes), metastasis, relapse in bone, and resistance to hormone therapy (Figure 4F and Table S2). Consistent with the upregulation of genes characteristic of the ERBB2 subtype, genes associated with the ERBB/KRAS/MEK/ERK pathway were also upregulated (Figure 4F). Notably, the hydrogel culture increased hypoxia-related genes and pluripotency genes, which are also suggestive of CSC-like phenotypes (Figure 4F). We examined whether the hydrogel-upregulated genes are associated with prognosis of breast cancer patients by analyzing large-scale RNA-seq datasets of 2509 human breast cancer patients [27,28]. The results showed that breast cancer patients harboring high expression of the hydrogel-upregulated genes have worse prognosis compared to other patients (Figure 4G). Collectively, the above global gene expression profiling and prognostic analyses suggest that culture on the poly(DMAAm-co-APTMA) hydrogel imposes more malignant, CSC-like phenotypes on breast cancer cells.

We then inquired whether treatment with RA induces expression of *HOXA5* and *ELF3* in MCF7 cells with elevated CSC properties. Treatment with RA suppressed proliferation of MCF7 cells cultured on poly(DMAAm-co-APTMA) gel and promoted expression of *HOXA5* and *ELF3* (Figure 4H). Moreover, treatment with a MEK inhibitor enhanced the RA-dependent induction of *HOXA5* and *ELF3* (Figure 4H). These results suggest that inhibition of ERK promotes ligand-dependent induction of RAR-target genes in breast cancer stem-like cells.

### 3.4. Treatment with RA and a MEK Inhibitor Synergistically Decreases Tumorigenicity of Breast Cancer Cells

**Figure 5:**
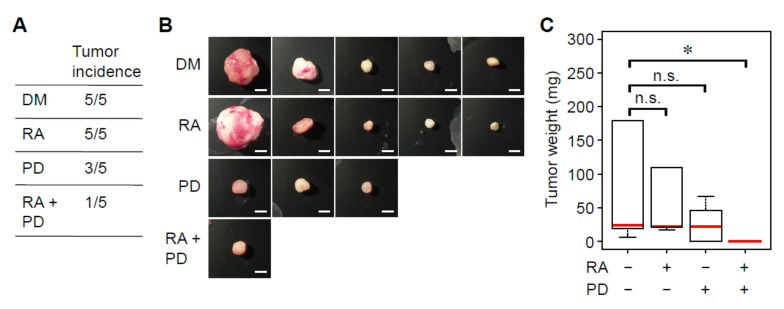
ERK inhibition and RAR activation synergistically decrease tumorigenicity of breast cancer cells. (**A**–**C**) Tumorigenicity of MDA-MB-231 breast cancer cells treated with or without RA and/or a MEK inhibitor (PD0325901) was evaluated by a tumor xenograft model in immunocompromised mice (*n* = 5). (**A**) The number of mice that developed tumors. (**B**) Representative images of the developed tumors. Scale bars: 4 mm. (**C**) Weight of tumors. *, *p* < 0.05 (Krukal-Wallis test followed by Wilcoxon rank sum tests). n.s.: not significant.

The synergistic effects of RA and a MEK inhibitor in inducing cell death and proliferation arrest encouraged us to inquire whether these two drugs synergistically suppress tumorigenic ability of breast cancer cells. To this end, we utilized an MDA-MB-231 tumor xenograft model in immunocompromised mice. In the control group, transplantation of MDA-MB-231 cells induced tumor formation in all mice tested (5 out of 5 mice) (Figure 5A,B). Treatment with a MEK inhibitor, but not RA, decreased tumorigenic ability of MDA-MB-231 cells (Figure 5A–C). Moreover, combined treatment with RA and a MEK inhibitor most strongly suppressed tumorigenic ability of MDA-MB-231 cells (Figure 5A–C). These results indicate that inhibition of the ERK pathway synergizes with retinoids to suppress tumorigenicity of breast cancer cells.

### 3.5. Suppression of RAR Signaling Coincides with Activation of ERK Signaling in Human Breast Cancers

**Figure 6:**
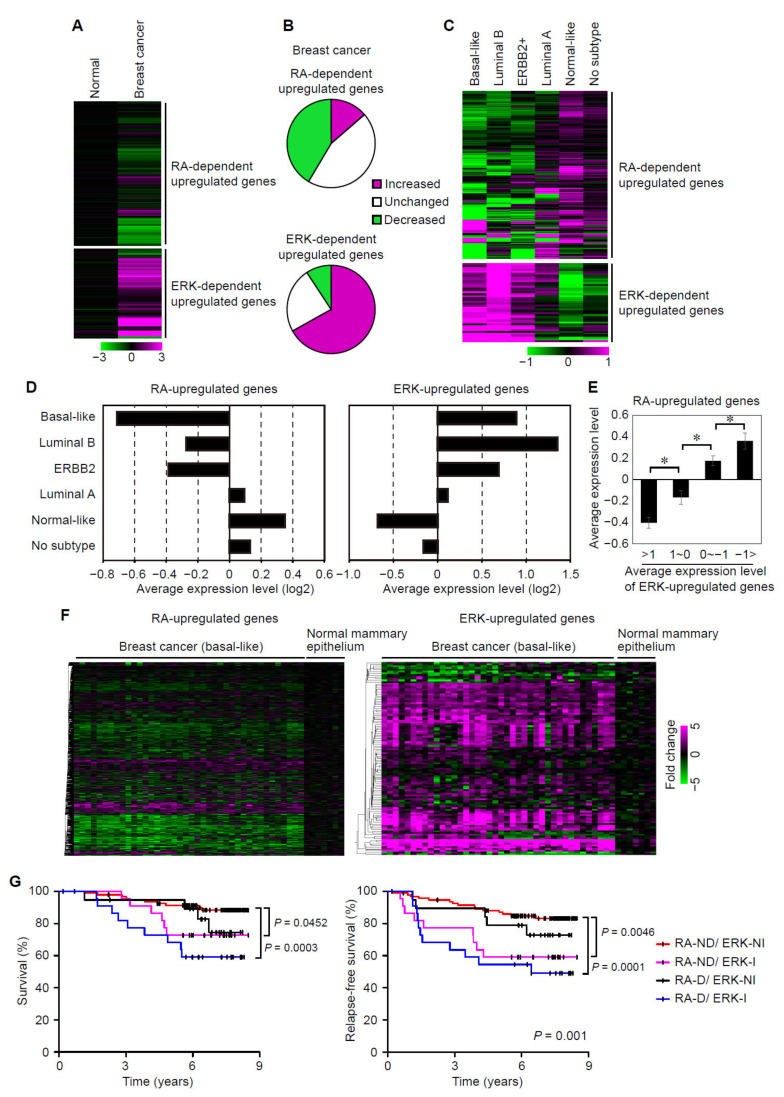
Expression profiles of RA- and ERK-target genes in human breast cancers. (**A**) Expression profiles of the RA- and ERK-dependent upregulated genes in normal mammary epithelia (*n* = 6) or breast cancers (*n* = 40) are shown. (**B**) The percentages of the RA- (Top) and ERK-dependent (bottom) upregulated genes whose expression levels in breast cancers are increased or decreased by more than 1.5-fold, as compared to those in normal mammary epithelia, are shown. (**C**) Expression profiles of the RA- and ERK-dependent upregulated genes in the indicated types of breast cancers are shown (Basal-like: *n* = 25, Luminal B: *n* = 23, ERBB2+: *n* = 15, Luminal A: 39, Normal-like: *n* = 37, No subtype: *n* = 20). (**D**) Average expression levels of RA-dependent and ERK-dependent upregulated genes in the indicated subtypes of breast cancers. (**E**) Average expression levels of RA-upregulated genes in patients with different expression levels of ERK-upregulated genes. *, *p* < 0.05 (one-way ANOVA followed by Tukey post hoc analyses). (**F**) Expression profiles of RA-dependent and ERK-dependent upregulated genes in individual basal-like breast cancers (*n* = 40) and normal mammary epithelia (*n* = 6) are shown. (**G**) Kaplan–Meier curves of survival (left) and relapse-free survival (right) among breast cancer patients (*n* = 159) are shown. The patient samples are classified in four categories based on expression profiles of the RA- and ERK-dependent upregulated genes (RA-ND/ERK-NI (*n* = 103), RA-ND/ERK-I (*n* = 19), RA-D/ERK-NI (*n* = 19), RA-D/ERK-I (*n* = 18)), as described. *p* values were obtained from log-rank tests.

Previous studies have shown that ERK signaling is often activated in many types of cancers through various mechanisms including mutations in *RAS* or *RAF*, overexpression of growth factor receptors, and promoted release of growth factors [19,20,21,45]. In breast cancers, estrogen has been shown to promote the release of heparan-bound epidermal growth factor (HB-EGF), thereby inducing activation of the EGF receptor and downstream ERK signaling [45]. Since our above results have suggested that ERK signaling activation suppresses RAR activity in breast cancer cells, we thought that downregulation of RAR signaling might coincides with activation of ERK signaling in breast cancers. To examine this idea, we analyzed the microarray datasets of human breast cancers [46] for both the RA-dependent upregulated genes and ERK-dependent upregulated genes, which were identified in cultured mammary epithelial cells [29,47]. Our analysis showed that the expression levels of a significant fraction of the RA-dependent upregulated genes are decreased whereas those of the ERK-dependent upregulated genes are increased in breast cancers, compared to normal mammary epithelia (Figure 6A,B). The percentages of the RA-dependent and the ERK-dependent upregulated genes, whose expression levels are decreased and increased, respectively, in breast cancers, are significantly higher than those of other genes (*p* < 0.001, Fisher’s exact test) (Figure 6B). This indicates that both RAR signaling suppression and ERK signaling activation occur in breast cancers. Notably, gene sets overrepresented in the ERK-upregulated genes included genes related to the E2F transcription factor and G2/M checkpoint (Table S3). The overlap of ERK-upregulated genes and cell cycle related E2F-target genes is consistent with the cell proliferation-promoting function of ERK. The gene sets overlapped with RA-upregulated genes included those related to estrogen responses, TNFA signaling, p53 signaling and apoptosis (Table S3). The overlaps with p53 signaling and apoptotic gene sets might account for anti-tumorigenic effects of RA on breast cancers.

Breast cancers could be classified into several distinct intrinsic subtypes that differ in their clinical outcomes [2,3,4,48]. We inquired which subtypes are accompanied by the above changes in RAR and ERK signaling. To this end, we analyzed the microarray datasets with information regarding subtypes of breast cancers and patients’ prognoses [49]. In three (basal-like, ERBB2+ and luminal B) out of six subtypes of breast cancers, the expression levels of most of the RA-dependent upregulated genes are decreased and those of the ERK-dependent upregulated genes are increased, as compared to those in other subtypes (luminal A, normal breast-like and no subtype) (Figure 6C). Indeed, the average expression level of RA-dependent upregulated genes was decreased, whereas that of ERK-dependent upregulated genes increased, in the basal-like, ERBB2+ and luminal B subtypes (Figure 6D). Importantly, patients with higher average expression levels of ERK-upregulated genes showed lower average expression levels of RA-upregulated genes (Figure 6E), indicating an inverse correlation between ERK- and RA-upregulated genes in breast cancer patients. Moreover, when analyzing individual patient data separately, decreased expression of RA-dependent upregulated genes and increased expression of ERK-dependent upregulated genes were observed in most of the patients with basal-like breast cancers (Figure 6F). These results suggest that ERK activation suppresses RAR signaling in specific subtypes of breast cancers.

### 3.6. Suppression of RAR Signaling and Activation of ERK Signaling Are Associated with Prognoses of Breast Cancer Patients

The above analyses revealed that suppression of RAR signaling and activation of ERK signaling occur in the three subtypes of breast cancers, basal-like, ERBB2+ and luminal B. Notably, it has been reported that patients with these three subtypes of breast cancers exhibit poorer prognoses than patients with other subtypes [2,3,4,48]. We thus speculated that the changes in RAR and ERK signaling correlates with prognoses of breast cancer patients. To examine the relationship between the patients’ prognoses and the changes in RAR and ERK signaling, we classified breast cancer patients into four categories (RA-ND/ERK-NI, RA-ND/ERK-I, RA-D/ERK-NI, and RA-D/ERK-I), based on expression profiles of the RA-dependent and ERK-dependent upregulated genes. RA-D (decreased) was defined as a sample, in which the number of the RA-dependent upregulated genes showing decreased expression (expression values < −0.5) was significantly larger than the average number in all samples, and other samples were defined as RA-ND (not decreased). ERK-I (increased) was defined as a sample, in which the number of the ERK-dependent upregulated genes showing increased expression (expression values > 1) was significantly larger than the average number in all samples, and other samples were defined as ERK-NI (not increased). Thus, we classified breast cancer patients into four categories: RA-ND/ERK-NI, RA-ND/ERK-I, RA-D/ERK-NI, and RA-D/ERK-I. Kaplan–Meier survival analysis of these breast cancer samples has shown that RA-ND/ERK-NI patients exhibit the best prognoses, such as the highest survival and relapse-free survival rates (Figure 6G). Conversely, RA-D/ERK-I patients exhibit the poorest prognoses (Figure 6G). Therefore, the concurrence of ERK signaling upregulation and RAR signaling downregulation correlates with poor prognoses, such as the higher risk of recurrence and the lower survival rate. Taken together, our results suggest that the ERK activation-induced suppression of RAR signaling would be associated with prognoses of several types of breast cancers.

### 3.7. Analyses of the Expression Status of ERK- and RA-Upregulated Genes in Large-Scale Gene Expression Datasets of Human Breast Cancers

**Figure 7:**
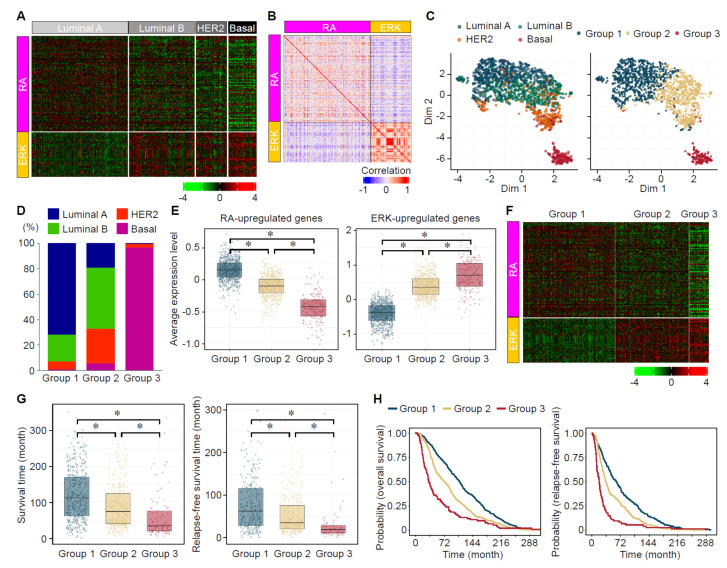
Expression profiles of RA- and ERK-target genes in human breast cancers. (**A**) Expression profiles of the RA- and ERK-dependent upregulated genes in major subtypes of breast cancers (Luminal A: *n* = 678, Luminal B: *n* = 461, HER2: *n* = 219, Basal: *n* = 199) are shown (*Z* scores). (**B**) Correlation matrix of the expression levels of the RA- and ERK-dependent upregulated genes in breast cancers (*n* = 1557). Pearson correlation coefficient of pairwise correlation of each gene was shown according to the color scale. (**C**) UMAP dimensionality reduction of breast cancer patients based on the expression levels of RA- and ERK-dependent upregulated genes. Patients were clustered into three groups (right), which were composed of different combination of breast cancer subtypes (left). (**D**) Proportion of breast cancer subtypes among the groups. (**E**) The average expression levels of the RA- and ERK-upregulated genes among the groups. *, *p* < 0.05 (Krukal-Wallis test followed by Wilcoxon rank sum test). (**F**) Expression profiles of the individual RA- and ERK-dependent upregulated genes among the groups. (**G**) Survival and relapse-free survival times of breast cancer patients among the groups. *, *p* < 0.05 (Krukal-Wallis test followed by Wilcoxon rank sum test). (**H**) (left) A Kaplan–Meier survival curve for breast cancer patients who died of the cancer (*n* = 933). (right) A relapse-free survival curve for the breast cancer patients who had the cancer recurrence (*n* = 643). Survival and relapse-free survival curves for all the patients are also provided in Figure S2. Statistical differences were observed between all possible pairs of survival and relapse-free survival curves by log-rank tests, with *p* values less than 0.05.

To further validate the consistency of our results obtained from breast cancer patient datasets, we analyzed expression profiles of ERK- and RA-upregulated genes at a larger scale with different human breast cancer gene expression datasets [27,28]. In these datasets, expression of RA-upregulated genes tended to be high in the luminal A and low in the basal subtypes (Figure 7A). On the contrary, expression of most ERK-upregulated genes was high in the basal and low in the luminal A subtypes (Figure 7A). The luminal B and HER2/ERBB2 subtypes showed intermediate expression levels for each of RA- and ERK- upregulated genes (see the detailed analysis below). (Figure 7A). Expression of most of RA-upregulated genes showed a clear inverse correlation with that of ERK-upregulated genes (Figure 7B). These results could support the idea that ERK activation suppresses RAR signaling in the specific subtypes of breast cancers.

A dimensionally reduction technique, Uniform Manifold Approximation and Projection (UMAP), was then performed on breast cancer patients based on the expression levels of RA- and ERK-upregulated genes (Figure 7C). In this low-dimensional space, the distribution of patient was gradually changing from the luminal A to the basal subtypes via the luminal B and HER2 subtypes. To examine the association between the expression status of the RA- and ERK-upregulated genes and patients’ prognoses, we performed k-means clustering on breast cancer patients into three categories from the set of RA- and ERK-upregulated genes. The distribution of these 3 groups was also visualized in UMAP space (Figure 7C). The group 1 was mostly composed of the luminal A and luminal B, whereas the group 3 was mainly composed of the basal subtype (Figure 7C,D). The group 2 included the luminal B, HER2, and luminal A subtypes, and a low percentage of the basal subtype (Figure 7C,D). The group 1 showed high average expression of RA-upregulated genes and low average expression of ERK-upregulated genes (Figure 7E). By contrast, the group 3 showed low average expression of RA-upregulated genes and high average expression of ERK-upregulated genes (Figure 7E). The group 2 showed intermediate average expression levels of RA- and ERK-upregulated genes (Figure 7E). This trend characterizing the 3 groups at the average expression level was also valid for a large proportion of individuals RA- and ERK-upregulated genes (Figure 7F). Analyses of the overall survival and relapse-free survival showed that the group 1 has the best, whereas the group 3 has the worst, prognoses (Figure 7G,H). Collectively, these data suggest that the expression status of RA- and ERK-upregulated genes has an inverse correlation, varies depending on the cancer subtypes, and is associated with prognoses of breast cancer patients.

## 4. Discussion

Despite the progress in our understanding of the pathological mechanisms and development of new therapeutic options, breast cancer remains the most common cancers and the leading cause of cancer-related death in women [1]. Since distinct subtypes of breast cancers harbor distinct genetic and epigenetic backgrounds and therefore exhibit different characteristics, development of therapeutic approaches suitable for individual subtypes is urgently needed to improve patient outcomes [2,3,4]. Although gene expression profiling has already determined the gene expression patterns that characterize individual subtypes [2,3,4], molecular mechanisms driving the gene expression programs have not been fully understood. Among potential anticancer drugs considered to be promising in breast cancer treatment are retinoids that act mainly through nuclear receptors, RARs and RXRs. As retinoids have been shown to suppress proliferation and induce cellular senescence and apoptosis in breast cancer cells, several clinical trials have been conducted [15]. The results of these trials, however, were not satisfactory due to the acquired resistance of cancer cells to retinoids [10,15]. In this study, we have shown that ERK signaling activation, which has been considered a common mechanism for many types of cancers, suppresses RAR signaling in breast cancer cells (Figure 1, Figure 2, Figure 3 and Figure 4). By contrast, pharmacological inhibition of ERK signaling augmented RAR signaling-mediated gene expression, cell proliferation arrest, apoptosis and suppression of tumorigenicity in breast cancer cells (Figure 1, Figure 2, Figure 3, Figure 4 and Figure 5). In line with these findings, ERK signaling activation is accompanied by suppression of RAR signaling in human breast cancers (Figure 6 and Figure 7). These results implicate ERK activation in retinoid resistance of breast cancer cells and suggest that ERK inhibition is key to enhancing therapeutic potential of retinoids in breast cancer patients.

It should be noted that the presence of CSCs underlies resistance to anti-cancer drugs and cancer recurrence in many types of cancers [40,41]. Thus, novel methods to eradicate CSCs are required for effective cancer treatment and prevention of recurrence. In this study, to examine the effects of RA and ERK inhibition on breast CSCs, we have established a method to induce CSC-like properties in MCF7 breast cancer cells (Figure 4). We showed that, by culturing cells on the poly(DMAAm-co-APTMA) hydrogel, expression of several CSC markers, such as *OCT4*, *NANOG*, and *CD44*, is induced within a few days (Figure 4D). Moreover, gene expression profiling revealed that gene sets relating to CSC-mediated processes, including cancer metastasis, relapse and drug resistance, are upregulated by the poly(DMAAm-co-APTMA) hydrogel (Figure 4E,F). Notably, the hydrogel-upregulated genes were associated with poor prognosis in breast cancer patients (Figure 4G)., These results suggest that cells rapidly acquire more malignant, CSC-like properties on this hydrogel. Importantly, RA treatment induced expression of potential tumor-suppressive genes, *HOXA5* and *ELF3*, also in the CSC-like cells (Figure 4H). Treatment with a MEK inhibitor significantly augmented the RA-dependent induction of these genes (Figure 4H). These results suggest that ERK activity regulates RAR signaling in breast CSC-like cells and that inhibition of ERK can improve sensitivity of breast CSCs to retinoid treatment. More generally, our results also suggest that rapid induction of CSC-like cells by the poly(DMAAm-co-APTMA) hydrogel would be useful to develop novel therapies effectively targeting CSCs.

Inhibition of RAR signaling and activation of ERK signaling are both hallmarks of many types of cancers, and therefore targeting these pathways have been considered a promising strategy for cancer treatment and prevention [9,10,16,19]. However, the relationship between RAR signaling inhibition and ERK signaling activation and its significance in cancer pathology remained unclear. In this study, we showed that downregulation of RAR signaling often coincides with upregulation of ERK signaling in human breast cancers (Figure 6 and Figure 7). Together with our previous study that showed antagonistic interactions between RAR and ERK signaling in colorectal cancer cells [17], ERK activation-induced suppression of RAR signaling might be a general mechanism for retinoid resistance in many types of cancers. In support of this notion, we also found that ERK activation suppresses RAR signaling in several other cell lines, such as HeLa cervical cancer cells, Caco-2 colon cancer cells, and HEK293 cells [17]. Thus, reversing the ERK-dependent suppression of RAR signaling seems to represent a novel strategy to overcome retinoid resistance in the treatment of many types of cancers.

It should be noted that our results have not demonstrated that retinoid therapy leads to ERK activation and thereby induces resistance to retinoids in breast cancer cells, like in the case of antiestrogen resistance following chronic antiestrogen exposure and estrogen deprivation [50]. Thus, although breast cancer cells harboring high ERK activity exhibit decreased RAR activity, it does not necessarily mean that this mechanism operates during retinoid therapy to confer therapy resistance on breast cancer cells. Nevertheless, our data have suggested that ERK inhibition would be key to enhancing therapeutic effects of retinoids on breast cancer cells harboring high ERK activity. Since ERK activation is among general mechanisms of many types of cancers, various combinations of the ERK pathway inhibitors and other anti-cancer drugs, such as conventional cytotoxic agents and inhibitors for other oncogenic pathways, have been long pursued [51,52]. Our findings provide a rationale for a novel combination of retinoids and ERK inhibition in breast cancer treatment. Notably, both ERK inhibition and retinoids have been reported to enhance efficacy of antiestrogen therapy [53,54]. Thus, combinatorial treatment of retinoids, ERK signaling inhibitors and antiestrogens might represent a novel strategy for breast cancer treatment.

Breast cancers have been classified into several subtypes according to their genetic and epigenetic properties [2,3,4]. In this study, we found that both ERK signaling activation and RAR signaling inhibition occur in the same set of subtypes, basal-like, HER2-enriched, and luminal B (Figure 6 and Figure 7). Moreover, RAR signaling inhibition and ERK signaling activation were observed in most of the individual basal-like breast cancer samples analyzed (Figure 6F). These results suggest that ERK signaling activation often suppresses RAR signaling in these subtypes of breast cancers. Importantly, the subtypes accompanied by the changes in ERK and RAR signaling have been shown to exhibit poorer prognosis than other subtypes [2,3,4]. These findings suggest that suppression of RAR signaling and activation of ERK signaling are associated with poor prognosis in breast cancer patients. Indeed, when breast cancer samples were classified based on the activation status of ERK and RAR signaling, patients with low RAR signaling activity and high ERK signaling activity showed the worst prognosis in terms of overall survival and relapse-free survival (Figure 6). Thus, RAR signaling inhibition and ERK signaling activation might play an important role in progression and therapeutic resistance of breast cancers. Further studies would be needed to reveal a molecular mechanism of the ERK-dependent inhibition of RAR activity and its role in breast cancers.

## 5. Conclusions

In conclusion, our results identify a novel mechanism of retinoid resistance in breast cancers and suggest that ERK inhibition might be key to overcoming the resistance. This provides crucial insights into the development of more effective and rational use of retinoids in breast cancer treatment. Moreover, our analyses also showed that changes in ERK and RAR signaling activity are associated with specific subtypes of breast cancers and affect patient prognosis. Thus, further elucidation of a role of these signaling pathways in breast cancers and development of therapeutic interventions targeting these pathways would be an important challenge in future studies.

## Data Availability

RNA-sequencing data of MCF7 cells cultured on the poly(DMAAm-co-APTMA) hydrogel can be downloaded from the DNA Data Bank of Japan (DDBJ) website (PRJDB12866).

## Supplementary Materials

The following supporting information can be downloaded at: https://www.mdpi.com/article/10.3390/cancers14235890/s1, Figure S1: Uncropped images of immunoblotting data; Figure S2: Kaplan-Meier curves of survival and relapse-free survival for all the breast cancer patients categorized into the three groups based on the RA- and ERK-upregulated genes; Table S1: Genes up- or down-regulated by the poly(DMAAm-co-APTMA) hydrogel; Table S2: Gene sets significantly upregulated by the poly(DMAAm-co-APTMA) hydrogel; Table S3: Gene sets significantly overlapped with RA- and ERK-upregulated genes.
