# Supplementary material for: ERK MAP Kinase Signaling Regulates RAR Signaling to Confer Retinoid Resistance on Breast Cancer Cells
Source: Cancers (Basel). 2022 Nov 29;14(23):5890. doi: 10.3390/cancers14235890 (PMC9739577; doi:10.3390/cancers14235890)

Figure S1. Uncropped images of immunoblotting data.

Figure 1A

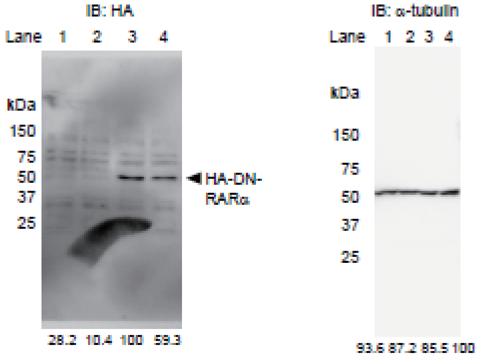

Figure 1B

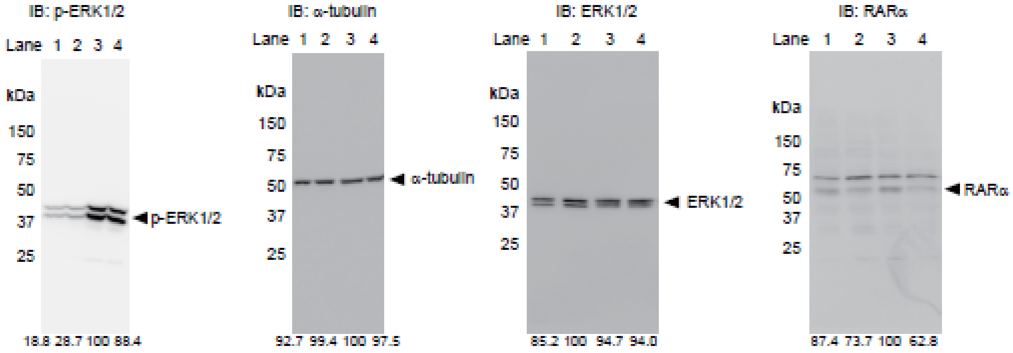

Figure 2A

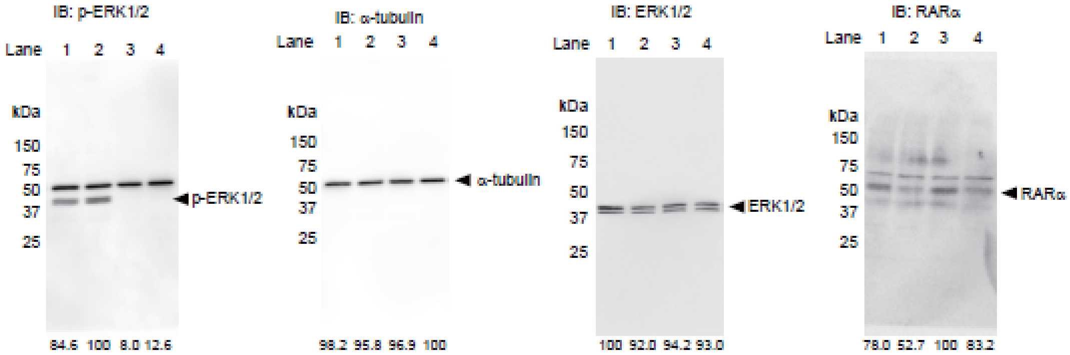

Figure S2. Kaplan-Meier curves of survival (left) and relapse-free survival (right) for all the breast cancer patients categorized into the three groups based on the RA- and ERK-upregulated genes.

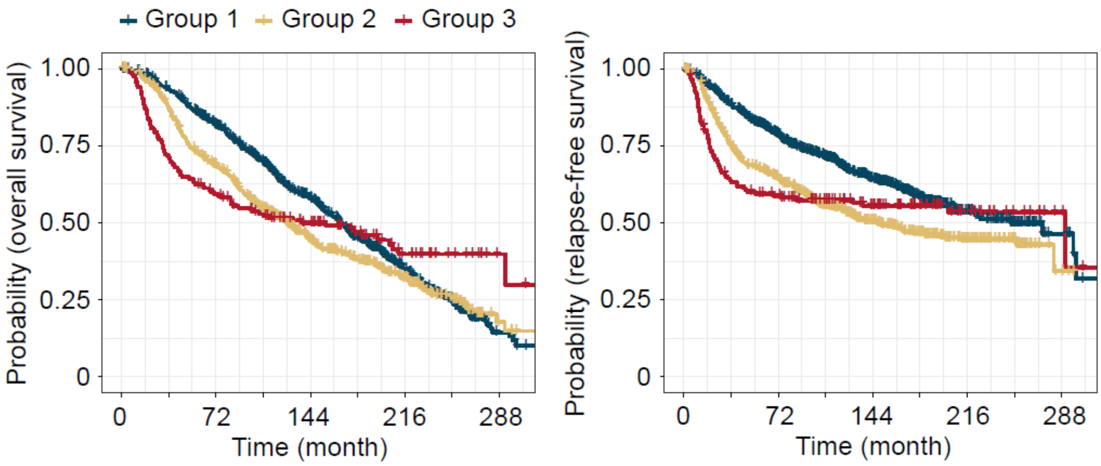

Supplement: Supplementary file 1 [file cancers-14-05890-s001.zip › Supplementary_material.pdf]
