# Supplementary material for: ERK MAP Kinase Signaling Regulates RAR Signaling to Confer Retinoid Resistance on Breast Cancer Cells
Source: Cancers (Basel). 2022 Nov 29;14(23):5890. doi: 10.3390/cancers14235890 (PMC9739577; doi:10.3390/cancers14235890)
Supplement: Supplementary file 1 [file cancers-14-05890-s001.zip › TableS1.pdf]

Table S1. Genes up- or down-regulated by the poly (DMAAm-co-APTMA) hydrogel

Upregulated genes

**Gene symbol**	**Fold change (log2) (PS vs Gel)**	***p*-value**
H2AC19	11.82147115	1.70953E-13
PYURF	8.475679867	3.41973E-08
H4C12	7.757044098	2.36136E-06
EGR4	7.068645966	2.30443E-05
TRIM39-RPP21	6.632918743	0.000140844
POTEJ	5.632486792	0.004818226
LBP	5.544325146	0.006437024
SLC13A4	5.003001088	0.027443888
KCNE4	4.715561639	0.048246457
FOSB	4.256055714	0.001780163
ITIH2	4.191536118	4.80845E-31
IL1R2	4.111999766	0.03612388
NCMAP	4.091780464	0.036645023
TCN1	3.997895582	0.043948191
MUC2	3.743302775	3.04229E-28
ABCC11	3.542396429	0.001182387
BPIFA2	3.503864526	0.009592407
GJC3	3.274000186	0.040392128
GP2	3.155385006	0.000158058
FOXA3	2.984205891	0.005185995
APOA1	2.926774365	0.019962862
SLC5A5	2.899785058	0.003743151
KCP	2.652457195	1.78477E-06
EGR2	2.603231962	1.97013E-05
PSAPL1	2.54766972	2.46315E-18
MTRNR2L3	2.511710161	8.95713E-08
FGB	2.465838643	0.000980323
CCRL2	2.416500601	0.043888878
TMEM45A	2.415680057	0.045754543
AOC1	2.347695744	0.004906034
CFB	2.292331365	0.000988397
PLAT	2.273991257	9.42217E-18
MUC5AC	2.258652048	2.07527E-26
HAAO	2.241183454	0.023033629
S100A7	2.100452391	0.001602938

HMGCS2	2.053549711	0.000341472
SLC44A4	2.036188463	0.026991565
EGR1	1.999849727	1.21365E-25
CD14	1.981910143	0.033019803
SLC25A18	1.976359451	0.027345148
AQP3	1.939706996	1.02096E-38
AGXT	1.933923922	0.008476058
CA9	1.875489961	0.001034359
KCNMA1	1.829835647	0.011813357
PTPRN2	1.815413449	5.65366E-18
SBSN	1.78987282	0.013767005
MTRNR2L6	1.782111126	1.0653E-06
AZGP1	1.779308224	5.49287E-18
TNFRSF11B	1.733567246	0.033387152
CEMIP	1.716596012	2.12197E-10
CCNP	1.711559669	2.10371E-17
GSTA1	1.693755265	0.014312379
PCOTH	1.668063074	0.002909773
IL32	1.65535413	0.027036656
GCNT2	1.638958959	0.029055101
TNFSF10	1.635239008	0.010395637
THEG	1.603836942	0.021148397
ATP6V0A4	1.601832669	4.29987E-06
UPK3A	1.59544316	3.49144E-05
MTRNR2L1	1.582241812	2.26438E-26
MTRNR2L8	1.57967652	5.70438E-32
CEACAM6	1.568961456	1.54605E-26
RASD1	1.539056286	2.93833E-10
NPC1L1	1.537909123	0.01001862
SYT13	1.52030608	1.12916E-07
APOD	1.512856047	3.58117E-12
CCL22	1.501348741	0.004472153
CEACAM5	1.501200756	1.1623E-27
SMPD3	1.493708577	8.05821E-05
UPK1B	1.490554167	0.019259179
BCL2L2-PABPN1	1.477243136	0.000147079
PARM1	1.477069223	2.38561E-10
KLK10	1.46553659	2.88328E-09
MSMB	1.464048906	8.24437E-07

CSTA	1.460629774	0.015456252
CXCL17	1.459125437	0.000460144
MTRNR2L2	1.457923099	1.76071E-23
SYNPO2L	1.457135295	0.027249913
CREB3L1	1.450341913	4.93885E-14
BPIFB1	1.445286739	4.73984E-07
SLC15A3	1.445054702	0.009712993
FBN1	1.444775329	9.22021E-06
SLC16A5	1.437716142	0.025947953
TSPAN1	1.428406379	2.15115E-12
SAMD9	1.422539402	0.020197846
WNK4	1.421596375	0.034276283
MAOA	1.417208543	1.58307E-10
LCN2	1.414999087	0.0255547
SDR16C5	1.406617405	4.23785E-05
AOX1	1.3916843	8.25087E-06
CHRM1	1.386643186	1.57638E-05
RNASE4	1.385708354	0.000629413
PTAFR	1.381033498	0.000190379
B3GNT6	1.379164943	0.026289411
ANG	1.376339723	0.000577891
DEPP1	1.365200611	0.004842496
OAS1	1.363723497	1.03602E-05
MTRNR2L9	1.351957264	1.44788E-08
WLS	1.339346414	0.037332608
C14orf132	1.335974102	1.44723E-16
SMIM22	1.315811874	0.002260533
SERPINA5	1.310599678	1.1642E-09
SPOCD1	1.296335197	0.002447652
TNFRSF19	1.286933227	0.010569694
KLK11	1.280664376	6.64916E-14
HSD17B11	1.2749528	0.00048649
PTPRH	1.267026276	0.000914601
ELAPOR1	1.266611705	2.45197E-19
FN1	1.253927793	3.44215E-14
SOX21	1.252090339	1.37484E-05
SDCBP2	1.247413189	0.027955792
ADCY5	1.24204469	1.30588E-11
SLIT1	1.231698959	0.032528138

NR4A1	1.229999682	0.013548417
C1orf116	1.21604708	0.001321517
IL2RB	1.212801482	0.005387866
EMP1	1.210160207	0.002538688
C2CD4A	1.202816698	0.004549039
MMP28	1.202571188	0.028770363
CCDC103	1.200097358	0.00157574
ANKRD35	1.195336097	0.00111648
TFF1	1.195245001	2.2197E-16
NDRG1	1.188259965	5.2303E-13
FOXD2	1.184404997	0.049234833
EPHB6	1.175497001	0.031315594
WDFY4	1.163979982	0.004617686
SPAG4	1.14724457	5.12332E-05
RIMBP3B	1.145969938	0.008699834
KLK13	1.135121972	0.041758286
SERPINA3	1.133996254	1.7937E-13
CXCR4	1.127599224	0.000607587
FUT3	1.122232361	0.032500784
CAPN9	1.121543821	5.0957E-06
AMTN	1.118311945	0.032796411
ITIH6	1.109155181	0.000706263
SERHL2	1.105077975	0.000582153
ETV4	1.089241479	3.07479E-05
CRAT	1.073738149	1.98371E-10
FAM161B	1.072199081	5.53949E-05
TNIK	1.072010007	0.000394267
CTSO	1.064714941	0.02983675
FBXO32	1.063966141	7.08168E-05
RAB27B	1.057004676	2.21212E-09
CD44	1.043705758	2.1116E-10
NUCB2	1.043434388	5.35248E-12
RGPD5	1.03966957	0.000373731
PRODH	1.035986463	0.028990554
S1PR3	1.03472788	5.55138E-10
AKAP12	1.018878691	0.02648715
LOC102724788	1.017682138	4.56235E-07
SHC4	1.015721742	2.91826E-06
DGLUCY	1.013088812	0.029555396

UGT2B15	1.006151069	0.000729387
TFPI2	1.003793196	0.00913227

Downregulated genes

Gene symbol	Fold change (log2) (PS vs Gel)	p-value
F8A2	-22.66606472	2.17288E-06
DNAJC25-GNG10	-6.773104292	0.032542623
PLGLB2	-5.573073699	0.005041588
GPR75-ASB3	-5.364290614	0.009630212
ABHD14A-ACY1	-5.017996286	0.022804238
CTAGE4	-4.914600653	0.000320662
RGPD6	-4.906215364	2.45804E-14
KCNAB3	-4.805437711	0.009266867
PDIA2	-4.170833602	0.037767728
RHBDL1	-3.449514385	3.31065E-05
TNFRSF25	-3.393755106	2.34893E-05
TPTE	-3.288525371	0.000596127
GOLGA8B	-3.273766179	0.001677558
GOLGA6L10	-3.114519261	0.00049746
C1QTNF3	-3.069200509	0.038213474
NDUFC2-KCTD14	-3.026235878	0.02949017
MAGED4B	-2.960786576	0.000294536
NPIPA2	-2.867738625	0.030029419
TRPV1	-2.797929658	0.001796467
FAM227A	-2.774544011	0.033735339
TNFRSF6B	-2.648792691	0.000415903
CYP24A1	-2.626340756	0.006333261
LOC101930307	-2.613522436	0.040978617
PVRIG	-2.608336079	0.014999655
PAQR6	-2.594494598	0.0003221
ZGLP1	-2.531562288	0.04838034
YJEFN3	-2.465440728	0.001511176
NPIPA8	-2.45372132	0.005597729
USP6	-2.449627867	0.044635766
LIME1	-2.429307931	7.42824E-09
PILRB	-2.367797251	0.045783373
GOLGA8A	-2.301393611	0.004818476
ZNF117	-2.276543596	0.006197333
AGAP9	-2.235246859	0.00151491

ATAD3B	-2.220189253	3.00913E-05
LCAT	-2.192587478	0.011228088
MMP19	-2.181092492	0.010648742
ZNF280C	-2.128169206	0.007398797
AGER	-2.109204366	0.003329893
BHLHB9	-2.105165606	0.023331156
ADAM8	-2.094790727	0.001794061
COL7A1	-2.08689165	0.036799492
IQCJ-SCHIP1	-2.079180719	0.011818666
AGAP5	-2.063077119	0.001058167
LRP5L	-2.049250459	0.030256052
TGFB2	-2.023266033	0.024427757
KIF18B	-2.015117622	8.17896E-13
RASL11A	-1.875700701	0.008719863
PABPC1L	-1.843250269	0.002441252
TRPC1	-1.807604949	0.016027026
MROH6	-1.806857279	3.34023E-09
ASTL	-1.793084266	0.046437581
CEP85L	-1.760577965	0.024239012
DLX2	-1.743964883	0.000953179
HCK	-1.74056765	7.32254E-05
SAP25	-1.734942663	0.000246472
C8orf44-SGK3	-1.731773919	0.011855612
TBC1D3L	-1.707946384	0.001392582
AGAP6	-1.66845853	0.000595021
CHD5	-1.656209731	0.000319651
HSF4	-1.64309869	0.00521305
NFKBIZ	-1.618606305	0.00080739
CPNE7	-1.61730058	0.049514442
ID3	-1.615771196	5.72474E-23
NR6A1	-1.610230954	0.000295584
RLN2	-1.59319139	0.021898335
L3MBTL1	-1.590022163	0.02902036
NPIPB6	-1.579174444	0.009701577
BRSK1	-1.567267045	0.010207465
NBPF11	-1.564102938	5.22176E-05
LOC101060341	-1.538997202	5.88709E-05
SPSB4	-1.535767012	0.000796024
NPIPB12	-1.530864172	5.57266E-09

BRICD5	-1.509519951	0.011165073
PTBP2	-1.508484753	0.002132968
GOLGA6L9	-1.507834424	0.003667576
LOC107984138	-1.496419772	0.022850821
NPIPB3	-1.445687203	1.87933E-09
SHISA2	-1.433881024	0.030684481
KCNMB3	-1.432331305	0.044895376
NBPF12	-1.430577781	8.07698E-05
CDH26	-1.430170068	0.000725616
RUNX2	-1.422286066	0.00870639
ID4	-1.391980876	1.5836E-09
KIF7	-1.391174629	9.0201E-05
PKDCC	-1.38275886	0.049853048
HMCN1	-1.378532945	1.15923E-18
HCN3	-1.378359226	0.010819489
RAB6B	-1.375948049	0.000125167
UHRF1	-1.360508527	1.14671E-12
OTUD3	-1.353449042	0.011786669
CCNL2	-1.343679677	0.041013843
NPIPB4	-1.339524767	0.009528667
HCN4	-1.336170979	0.021668494
SMIM32	-1.323354356	0.014786861
PPFIA4	-1.322972511	0.008858245
LTB4R2	-1.318664229	0.021101141
SCML2	-1.317206666	0.010303128
SCX	-1.311526759	0.00047336
EVPLL	-1.304285643	0.028366935
ANKRD6	-1.296601156	0.001898889
ID1	-1.296357513	1.66575E-19
PRRT1B	-1.293471891	1.9006E-07
CCDC85A	-1.277089489	2.28549E-06
NPIPA1	-1.275195886	0.003973534
OLFML2A	-1.267050869	2.73012E-05
MYSM1	-1.26669652	0.009837388
ID2	-1.259474931	3.08594E-14
KCNQ4	-1.258738793	0.002482504
TGM1	-1.253352156	0.020250513
LOC107986800	-1.248928328	0.001448011
CCDC84	-1.248202921	0.024825916

BCL11B	-1.244958491	0.005024351
PBX4	-1.244834172	0.026604115
NPIPB5	-1.241768324	0.008662588
PTPRS	-1.24153897	4.28111E-15
CCN5	-1.232548741	3.82816E-09
SEMA6A	-1.231037096	0.008475716
LPIN1	-1.197443237	5.54558E-05
NFKBID	-1.194940066	0.005045235
MAST1	-1.192311567	0.000375281
MDGA1	-1.180456576	0.041899507
PIF1	-1.177288243	0.002423724
IGFBP3	-1.173197442	0.03688607
C6orf141	-1.171502068	0.000213066
WDR27	-1.170457317	0.037112099
FAM193B	-1.168886567	1.79987E-06
LIN7B	-1.168856922	0.000374043
MTX3	-1.16616128	0.011743755
AMH	-1.155571899	1.2401E-06
IRGQ	-1.152998345	0.006929652
ZNF418	-1.151590681	0.036760645
L1CAM	-1.144366828	5.51692E-10
UGT1A6	-1.13204212	6.8634E-07
VAMP1	-1.128572421	0.03852104
PIK3CD	-1.12252552	0.032802007
GPC2	-1.114900726	0.030594413
CCM2L	-1.114217384	0.00289637
DCUN1D2	-1.113595659	0.009614404
FAM131C	-1.108964214	0.005800177
MAMDC4	-1.10065876	0.039003234
ADGRA2	-1.096415449	0.011019593
ANKS3	-1.093426386	0.001655939
SMAD6	-1.086911768	8.2349E-06
ZIC5	-1.086811902	0.037978858
CARMIL2	-1.080909979	0.009082126
DNMT3B	-1.073359969	3.46418E-08
ADM5	-1.071892901	0.007558682
NBPF19	-1.069684723	4.99172E-07
NBPF20	-1.066586729	0.000703502
CREBZF	-1.065244967	1.679E-06

TCF7L2	-1.064969656	0.000542889
GINS1	-1.063624593	0.000232582
TPM2	-1.061160854	0.034698734
ADAMTS7	-1.053839455	0.016474607
SYNM	-1.053278844	0.001851129
RAP1GAP2	-1.049599661	0.000691767
MAPK8IP3	-1.049534282	3.5149E-06
CHN1	-1.047312061	0.000262502
GRAMD4	-1.046391332	1.09406E-05
AHRR	-1.045408437	0.000903895
SYNDIG1	-1.040202554	0.014175629
LOC101928841	-1.036554933	0.00479866
NANOS1	-1.034928097	0.015561663
NPIPB13	-1.034672641	0.021378999
GLS	-1.033587282	0.000342013
MEX3D	-1.030567742	3.06216E-08
DFFB	-1.020675466	0.026570829
MCC	-1.016185733	0.011846058
JPH1	-1.01362885	0.000657751
CACNB1	-1.01304857	0.030860039
TMEM41B	-1.007813535	0.027618632
CCDC134	-1.004811281	0.007859901
