# Supplementary material for: ERK MAP Kinase Signaling Regulates RAR Signaling to Confer Retinoid Resistance on Breast Cancer Cells
Source: Cancers (Basel). 2022 Nov 29;14(23):5890. doi: 10.3390/cancers14235890 (PMC9739577; doi:10.3390/cancers14235890)
Supplement: Supplementary file 1 [file cancers-14-05890-s001.zip › TableS2.pdf]

Table S2. Gene sets significantly upregulated by the poly (DMAAm-co-APTMA) hydrogel

Curated gene sets

Gene sets	NES	NOM p-value	FDR q-value
DOANE_BREAST_CANCER_CLASSES_UP	2.6299665	0	0
SMID_BREAST_CANCER_ERBB2_UP	2.545426	0	0
HSIAO_LIVER_SPECIFIC_GENES	2.5395331	0	0
KEGG_COMPLEMENT_AND_COAGULATION_CASCADES	2.474672	0	0
ONDER_CDH1_TARGETS_3_DN	2.4616623	0	0
PEDERSEN_METASTASIS_BY_ERBB2_ISOFORM_4	2.440284	0	0
CROMER_TUMORIGENESIS_DN	2.41769	0	0
WP_PEPTIDE_GPCRS	2.413965	0	0
SENGUPTA_NASOPHARYNGEAL_CARCINOMA_DN	2.3874524	0	0
HUPER_BREAST_BASAL_VS_LUMINAL_UP	2.3605404	0	0
KEGG_LYSOSOME	2.3523505	0	0
LIN_SILENCED_BY_TUMOR_MICROENVIRONMENT	2.3502805	0	0
WP_COMPLEMENT_AND_COAGULATION_CASCADES	2.3464363	0	0
MOHANKUMAR_HOXA1_TARGETS_DN	2.345415	0	0
LIU_VAV3_PROSTATE_CARCINOGENESIS_UP	2.3442726	0	0
REACTOME_INTERLEUKIN_10_SIGNALING	2.2972586	0	6.856E-05
HOSHIDA_LIVER_CANCER_SUBCLASS_S3	2.2892535	0	6.453E-05
REACTOME_SRP_DEPENDENT_COTRANSLATIONAL_PROTEIN_TARGETING_TO_MEMBRANE	2.2855177	0	6.094E-05
KRASNOSELSKAYA_ILF3_TARGETS_UP	2.2786486	0	5.774E-05
MCLACHLAN_DENTAL_CARIES_UP	2.2707064	0	5.485E-05
BOYLAN_MULTIPLE_MYELOMA_PCA1_UP	2.2658894	0	5.224E-05
HECKER_IFNB1_TARGETS	2.2480822	0	9.874E-05
BOSCO_EPITHELIAL_DIFFERENTIATION_MODULE	2.2110972	0	0.000334
APPEL_IMATINIB_RESPONSE	2.2108233	0	0.0003201
CAIRO_LIVER_DEVELOPMENT_DN	2.2059321	0	0.0003502
PEDERSEN_METASTASIS_BY_ERBB2_ISOFORM_6	2.1962574	0	0.0003367
MISSIAGLIA_REGULATED_BY_METHYLATION_UP	2.1959393	0	0.0003242

VECCHI_GASTRIC_CANCER_ADVANCED_VS_EARLY_DN	2.1909978	0	0.0003517
WP_FIBRIN_COMPLEMENT_RECEPTOR_3_SIGNALING_PATHWAY	2.1874168	0	0.0003396
REACTOME_NEUTROPHIL_DEGRANULATION	2.1873872	0	0.0003282
WANG_BARRETTS_ESOPHAGUS_AND_ESOPHAGUS_CANCER_UP	2.185738	0	0.0003176
RICKMAN_HEAD_AND_NECK_CANCER_D	2.1791682	0	0.000409
SANA_RESPONSE_TO_IFNG_UP	2.1678226	0	0.0006294
BLANCO_MELO_RESPIRATORY_SYNCYTIAL_VIRUS_INFECTION_A594_CELLS_UP	2.1676319	0	0.0006109
REACTOME_DEFECTIVE_C1GALT1C1_CAUSES_TN_POLYAGGLUTINATION_SYNDROME_TNPS	2.1547022	0	0.0006549
REACTOME_EUKARYOTIC_TRANSLATION_ELONGATION	2.1532986	0	0.0006675
REACTOME_ANTIMICROBIAL_PEPTIDES	2.15258	0	0.0006494
KUROZUMI_RESPONSE_TO_ONCOCYTIC_VIRUS	2.1489928	0	0.0006609
REACTOME_TERMINATION_OF_O_GLYCAN_BIOSYNTHESIS	2.1479154	0	0.0006439
ZHONG_SECRETOME_OF_LUNG_CANCER_AND_MACROPHAGE	2.1455925	0	0.0006278
BENNETT_SYSTEMIC_LUPUS_ERYTHEMATOSUS	2.1444566	0	0.0006125
SARRIO_EPITHELIAL_MESENCHYMAL_TRANSITION_DN	2.143198	0	0.0006236
SMID_BREAST_CANCER_RELAPSE_IN_BONE_UP	2.1380198	0	0.0007365
LIANG_SILENCED_BY_METHYLATION_2	2.1349673	0	0.0007451
BILANGES_SERUM_AND_RAPAMYCIN_SENSITIVE_GENES	2.1311007	0	0.0007777
BLANCO_MELO_COVID19_SARS_COV_2_POS_PATIENT_LUNG_TISSUE_UP	2.1256933	0	0.0009279
KEGG_RIBOSOME	2.1200156	0	0.0010693
DOANE_BREAST_CANCER_ESR1_UP	2.1095352	0	0.0012056
NABA_ECM_REGULATORS	2.1078854	0	0.0012481
ANDERSEN_CHOLANGIOCARCINOMA_CLASS2	2.1074522	0	0.0012451
REACTOME_O_LINKED_GLYCOSYLATION_OF_MUCINS	2.0808702	0	0.002314
REACTOME_FORMATION_OF_FIBRIN_CLOT_CLOTTING_CASCADE	2.077708	0	0.0024382
WP_FOLATE_METABOLISM	2.0775218	0	0.0023922
NAKAMURA_METASTASIS_MODEL_DN	2.0753367	0	0.002409
REACTOME_COMPLEMENT_CASCADE	2.0735288	0	0.0024251
ONDER_CDH1_TARGETS_2_DN	2.0707185	0	0.0024603
DEMAGALHAES_AGING_UP	2.0681553	0	0.0025123

LEE_LIVER_CANCER_CIPROFIBRATE_UP	2.0578966	0	0.0030915
REACTOME_SLC_MEDIATED_TRANSMEMBRANE_TRANSPORT	2.0578482	0	0.0030391
BLANCO_MELO_COVID19_SARS_COV_2_INFECTION_CALU3_CELLS_UP	2.0537763	0	0.0031705
PEDERSEN_METASTASIS_BY_ERBB2_ISOFORM_1	2.053008	0	0.0032608
GILDEA_METASTASIS	2.0527084	0	0.0032082
JINESH_BLEBBISHIELD_TRANSFORMED_STEM_CELL_SPHERES_UP	2.0479884	0	0.0033639
COLIN_PILOCYTIC_ASTROCYTOMA_VS_GLIOBLASTOMA_UP	2.0466928	0	0.0033972
LIEN_BREAST_CARCINOMA_METAPLASTIC_VS_DUCTAL_DN	2.0452902	0	0.0034462
REACTOME_RESPONSE_TO_ELEVATED_PLATELET_CYTOSOLIC_CA2	2.0431786	0	0.0035109
WP_VITAMIN_B12_METABOLISM	2.0327756	0.002132196	0.0040301
BOYAULT_LIVER_CANCER_SUBCLASS_G56_DN	2.031753	0	0.0040512
REACTOME_IRAK4_DEFICIENCY_TLR2_4	2.0182216	0.002105263	0.0052094
ELVIDGE_HIF1A_AND_HIF2A_TARGETS_DN	2.0176268	0	0.0052122
REACTOME_EUKARYOTIC_TRANSLATION_INITIATION	2.0163784	0	0.0052615
SERVITJA_ISLET_HNF1A_TARGETS_DN	2.0142512	0	0.0053854
LIM_MAMMARY_STEM_CELL_DN	2.0124516	0	0.0054164
DELPUECH_FOXO3_TARGETS_UP	2.0114164	0	0.0054911
ANDERSEN_LIVER_CANCER_KRT19_DN	2.0095577	0	0.0056207
PID_INTEGRIN2_PATHWAY	2.0068643	0	0.0057764
REACTOME_SELENOAMINO_ACID_METABOLISM	2.0057683	0	0.0058435
CAVARD_LIVER_CANCER_MALIGNANT_VS_BENIGN	2.0043347	0	0.0059084
RIGGINS_TAMOXIFEN_RESISTANCE_UP	1.99816	0	0.0063744
WANG_METASTASIS_OF_BREAST_CANCER_ESR1_DN	1.9980518	0	0.0062947
ZHANG_INTERFERON_RESPONSE	1.9922775	0	0.0067946
COLDREN_GEFITINIB_RESISTANCE_DN	1.9885302	0	0.0070178
REACTOME_CHEMOKINE_RECEPTORS_BIND_CHEMOKINES	1.9862468	0	0.007222
MCDOWELL_ACUTE_LUNG_INJURY_UP	1.984051	0.002105263	0.0074086
WP_BLOOD_CLOTTING_CASCADE	1.9814872	0	0.0076283
WP_GLYCOSYLATION_AND_RELATED_CONGENITAL_DEFECTS	1.9797422	0	0.0076662
BLANCO_MELO_HUMAN_PARAINFLUENZA_VIRUS_3_INFECTION_A594_CELLS_UP	1.9792496	0	0.007641

SENGUPTA_NASOPHARYNGEAL_CARCINOMA_WITH_LMP1_DN	1.9740973	0	0.0081379
LEE_LIVER_CANCER_ACOX1_UP	1.9738998	0	0.0080709
BIOCARTA_TFF_PATHWAY	1.9708114	0	0.0082465
WAGNER_APO2_SENSITIVITY	1.9695928	0	0.0083593
KIM_LRRC3B_TARGETS	1.967382	0	0.0085518
XIE_ST_HSC_S1PR3_OE_UP	1.9667645	0	0.0085416
BARIS_THYROID_CANCER_DN	1.9667482	0	0.0084624
WU_SILENCED_BY_METHYLATION_IN_BLADDER_CANCER	1.9663618	0	0.0084199
BLANCO_MELO_COVID19_SARS_COV_2_INFECTION_A594_ACE2_EXPRESSING_CELLS_RUXOLITINIB_DN	1.9662352	0	0.0083322
ROY_WOUND_BLOOD_VESSEL_DN	1.9662112	0	0.0082463
ELVIDGE_HYPOXIA_BY_DMOG_UP	1.9650836	0	0.00824
ELVIDGE_HYPOXIA_UP	1.9619198	0	0.008486
REACTOME_REGULATION_OF_TLR_BY_ENDOGENOUS_LIGAND	1.9593879	0	0.00865
WILENSKY_RESPONSE_TO_DARAPLADIB	1.9581206	0	0.0087379
JOSEPH_RESPONSE_TO_SODIUM_BUTYRATE_UP	1.9439317	0	0.0106968
ZHANG_ANTIVIRAL_RESPONSE_TO_RIBAVIRIN_UP	1.942997	0.00204918	0.0107302
ODONNELL_TARGETS_OF_MYC_AND_TFRC_UP	1.9422415	0	0.0107311
WENG_POR_TARGETS_LIVER_DN	1.939987	0	0.0110353
WP_CYTOPLASMIC_RIBOSOMAL_PROTEINS	1.9391725	0	0.0110036
NABA_MATRISOME_ASSOCIATED	1.9383389	0	0.0110331
FUJII_YBX1_TARGETS_UP	1.9380156	0	0.0110017
CHIANG_LIVER_CANCER_SUBCLASS_PROLIFERATION_DN	1.936174	0	0.0111908
REACTOME_TRANSPORT_OF_INORGANIC_CATIONS_ANIONS_AND_AMINO_ACIDS_OLIGOPEPTIDES	1.9341414	0	0.0113563
WP_GLYCOLYSIS_AND_GLUCONEOGENESIS	1.9332935	0	0.0114115
ELVIDGE_HIF1A_TARGETS_DN	1.9282204	0	0.0120998
SMID_BREAST_CANCER_RELAPSE_IN_PLEURA_DN	1.9271084	0.00212766	0.0121281
ZHENG_IL22_SIGNALING_UP	1.9269191	0	0.012031
PAPASPYRIDONOS_UNSTABLE_ATEROSCLEROTIC_PLAQUE_UP	1.9248494	0	0.0122861
WP_HUMAN_COMPLEMENT_SYSTEM	1.9245589	0	0.012227
TARTE_PLASMA_CELL_VS_B_LYMPHOCYTE_UP	1.9233407	0	0.0124127

HINATA_NFKB_TARGETS_KERATINOCYTE_UP	1.919058	0	0.0129174
FLECHNER_BIOPSY_KIDNEY_TRANSPLANT_REJECTED_VS_OK_UP	1.9178106	0	0.0130831
BOWIE_RESPONSE_TO_TAMOXIFEN	1.9174716	0	0.0130375
GREENBAUM_E2A_TARGETS_DN	1.9170926	0	0.0129567
WESTON_VEGFA_TARGETS	1.913691	0	0.0134299
REACTOME_PEPTIDE_LIGAND_BINDING_RECEPTORS	1.9129725	0	0.0134185
STEGER_ADIPOGENESIS_DN	1.9129474	0.002164502	0.0133102
REACTOME_MATURATION_OF_SARS_COV_2_SPIKE_PROTEIN	1.9122106	0	0.0133604
GROSS_HYPOXIA_VIA_ELK3_DN	1.9099122	0	0.0137052
RICKMAN_HEAD_AND_NECK_CANCER_E	1.9089155	0	0.0138301
WP_CORI_CYCLE	1.9085906	0.002114165	0.0137559
WP_IL1_AND_MEGAKARYOCYTES_IN_OBESITY	1.907347	0.002066116	0.0138427
LENAOUR_DENDRITIC_CELL_MATURATION_DN	1.904063	0	0.0144401
PID_UPA_UPAR_PATHWAY	1.9037852	0	0.0143885
WP_COMPUTATIONAL_MODEL_OF_AEROBIC_GLYCOLYSIS	1.9037336	0	0.0143044
YAMASHITA_LIVER_CANCER_STEM_CELL_DN	1.9022356	0	0.0143613
SCHUETZ_BREAST_CANCER_DUCTAL_INVASIVE_DN	1.8997484	0	0.0148965
KEGG_TRYPTOPHAN_METABOLISM	1.8882023	0	0.0170402
ZHONG_SECRETOME_OF_LUNG_CANCER_AND_FIBROBLAST	1.8879836	0	0.0169549
SANA_TNF_SIGNALING_UP	1.885562	0.002197802	0.0173406
REACTOME_POST_TRANSLATIONAL_MODIFICATION_SYNTHESIS_OF_GPI_ANCHORED_PROTEINS	1.8852066	0	0.0173098
KEGG_PROTEIN_EXPORT	1.8837197	0.002197802	0.0174285
WENG_POR_TARGETS_GLOBAL_DN	1.8832507	0	0.0173977
JOHNSTONE_PARVB_TARGETS_2_UP	1.8827304	0	0.0173519
PID_NFAT_TFPATHWAY	1.8786004	0.002150538	0.0181503
REACTOME_SPHINGOLIPID_DE_NOVO_BIOSYNTHESIS	1.878009	0.002016129	0.018206
REACTOME_INSULIN_RECEPTOR_RECYCLING	1.874612	0	0.0187609
CHARAFE_BREAST_CANCER_BASAL_VS_MESENCHYMAL_UP	1.8744605	0	0.0186689
DOANE_RESPONSE_TO_ANDROGEN_UP	1.8742765	0	0.0185932
ABE_VEGFA_TARGETS_30MIN	1.872747	0	0.018815

LINDSTEDT_DENDRITIC_CELL_MATURATION_D	1.872141	0.002016129	0.0188418
REACTOME_SPHINGOLIPID_METABOLISM	1.8703218	0	0.0191104
REACTOME_COMMON_PATHWAY_OF_FIBRIN_CLOT_FORMATION	1.867742	0.004132231	0.0195728
BLANCO_MELO_COVID19_BRONCHIAL_EPITHELIAL_CELLS_SARS_COV_2_INFECTION_UP	1.8676628	0	0.0194578
WP_SELENIUM_MICRONUTRIENT_NETWORK	1.864195	0.002087683	0.0201327
GRANDVAUX_IFN_RESPONSE_NOT_VIA_IRF3	1.8635348	0.002141328	0.0201362
RUTELLA_RESPONSE_TO_HGF_VS_CSF2RB_AND_IL4_UP	1.8624605	0	0.0202038
REACTOME_O_LINKED_GLYCOSYLATION	1.8581941	0	0.021093
HELLEBREKERS_SILENCED_DURING_TUMOR_ANGIOGENESIS	1.8552474	0	0.0216641
MOSERLE_IFNA_RESPONSE	1.8491564	0	0.0230913
BROWN_MYELOID_CELL_DEVELOPMENT_UP	1.8489788	0	0.0229868
OHGUCHI_LIVER_HNF4A_TARGETS_UP	1.847793	0	0.0231443
BROWNE_HCMV_INFECTION_2HR_UP	1.8477287	0.006289308	0.0230065
CREIGHTON_AKT1_SIGNALING_VIA_MTOR_DN	1.8438833	0.002183406	0.0237658
BIOCARTA_AMI_PATHWAY	1.8435512	0.002016129	0.0236933
TARTE_PLASMA_CELL_VS_PLASMABLAST_UP	1.8433173	0	0.0236489
WP_GPCRS_CLASS_A_RHODOPSINLIKE	1.8431516	0	0.023538
SMID_BREAST_CANCER_RELAPSE_IN_LUNG_DN	1.8389763	0.002008032	0.0245058
WESTON_VEGFA_TARGETS_3HR	1.8372169	0	0.0248575
FARDIN_HYPOXIA_11	1.8368168	0.002183406	0.0248069
HSIAO_HOUSEKEEPING_GENES	1.8336359	0	0.0257487
WP_TRYPTOPHAN_METABOLISM	1.8318194	0.00422833	0.0261268
KEGG_HISTIDINE_METABOLISM	1.8307868	0.003976143	0.0262675
RUTELLA_RESPONSE_TO_HGF_UP	1.8289618	0	0.0266741
REACTOME_BIOSYNTHESIS_OF_THE_N_GLYCAN_PRECURSOR_DOLICHOL_LIPID_LINKED_OLIGOSACCHARIDE_LLO_AND_TRANSFER_TO_A_NASCENT_PROTEIN	1.8281268	0	0.0267153
REACTOME_DECTIN_2_FAMILY	1.8273399	0.004246285	0.0267886
REACTOME_INTERFERON_ALPHA_BETA_SIGNALING	1.8271118	0	0.0267289
SMID_BREAST_CANCER_RELAPSE_IN_BRAIN_DN	1.8242095	0.002061856	0.0276049
KEGG_VIBRIO_CHOLERAE_INFECTION	1.8237619	0	0.0275972
REACTOME_FERTILIZATION	1.8236148	0	0.027466

NAKAJIMA_MAST_CELL	1.8232684	0.004357298	0.0273795
CHARAFE_BREAST_CANCER_LUMINAL_VS_MESENCHYMAL_UP	1.8189963	0	0.0285682
TIEN_INTESTINE_PROBIOTICS_24HR_DN	1.8163261	0	0.0292467
CHANG_IMMORTALIZED_BY_HPV31_DN	1.8146586	0	0.0296637
CHEOK_RESPONSE_TO_MERCAPTOPURINE_DN	1.8094089	0.002178649	0.0311609
DAUER_STAT3_TARGETS_DN	1.8092616	0.002012072	0.0310085
TSAI_RESPONSE_TO_RADIATION_THERAPY	1.8081014	0.006122449	0.0312189
VANDESLUIS_COMMD1_TARGETS_GROUP_3_UP	1.8069416	0.001976285	0.0313864
TIAN_BHLHA15_TARGETS	1.8042996	0.004115226	0.0319919
NAKAYAMA_SOFT_TISSUE_TUMORS_PCA1_UP	1.8041657	0.004535148	0.0318682
STEARMAN_TUMOR_FIELD_EFFECT_UP	1.804142	0.0041841	0.0317045
REACTOME_PLASMA_LIPOPROTEIN_CLEARANCE	1.8036507	0.004132231	0.0316758
BECKER_TAMOXIFEN_RESISTANCE_UP	1.8026596	0.00210084	0.0319329
MEBARKI_HCC_PROGENITOR_WNT_UP_CTNNB1_INDEPENDENT	1.7984971	0.012738854	0.0332767
SU_LIVER	1.7966913	0.002053388	0.0337279
KEGG_RIBOFLAVIN_METABOLISM	1.7945447	0.004175365	0.0342143
DELYS_THYROID_CANCER_UP	1.7944449	0	0.0340769
REACTOME_DISEASES_ASSOCIATED_WITH_O_GLYCOSYLATION_OF_PROTEINS	1.7936753	0.002040816	0.0341662
ZHENG_FOXP3_TARGETS_UP	1.7928536	0.004273505	0.034381
JAEGER_METASTASIS_DN	1.7916855	0	0.0346824
REACTOME_INTERLEUKIN_4_AND_INTERLEUKIN_13_SIGNALING	1.7908906	0.00210084	0.034772
REACTOME_ACTIVATION_OF_THE_MRNA_UPON_BINDING_OF_THE_CAP_BINDING_COMPLEX_AND_EIFS_AND_SUBSEQUENT_BINDING_TO_43S	1.7904652	0	0.0347613
REACTOME_SARS_COV_2_INFECTION	1.787979	0	0.0356067
REACTOME_DISEASES_ASSOCIATED_WITH_N_GLYCOSYLATION_OF_PROTEINS	1.7877461	0.001988072	0.0355056
WU_CELL_MIGRATION	1.7839221	0	0.0368764
KORKOLA_TERATOMA_UP	1.7803221	0.004329004	0.0381409
BURTON_ADIPOGENESIS_8	1.7798847	0	0.0381625
PLASARI_NFIC_TARGETS_BASAL_UP	1.7784662	0.010060363	0.0384655
SPIRA_SMOKERS_LUNG_CANCER_UP	1.7769481	0.010683761	0.0388831
BRACHAT_RESPONSE_TO_METHOTREXATE_DN	1.7755489	0.004273505	0.0393377

JINESH_BLEBBISHIELD_VS_LIVE_CONTROL_DN	1.7739702	0	0.0397046
SALVADOR_MARTIN_PEDIATRIC_TBD_ANTI_TNF_THERAPY_NONRESPONDER_POST_TREATMENT_UP	1.7737939	0.010438413	0.0395723
ZHOU_INFLAMMATORY_RESPONSE_LPS_UP	1.7727582	0.002132196	0.0398048
MENSE_HYPOXIA_UP	1.7715067	0	0.0401175
WIELAND_UP_BY_HBV_INFECTION	1.7705755	0.002159827	0.0402681
CHIARADONNA_NEOPLASTIC_TRANSFORMATION_CDC25_DN	1.7699796	0	0.0402892
WEST_ADRENOCORTICAL_TUMOR_MARKERS_DN	1.7690269	0.002066116	0.0405549
KIM_LIVER_CANCER_POOR_SURVIVAL_DN	1.7684075	0.014799154	0.0406244
NABA_MATRISOME	1.7680725	0	0.0405677
TIEN_INTESTINE_PROBIOTICS_6HR_UP	1.7679032	0	0.0404109
YAO_TEMPORAL_RESPONSE_TO_PROGESTERONE_CLUSTER_9	1.7673994	0	0.0404106
KEGG_TYPE_I_DIABETES_MELLITUS	1.7643201	0.008791209	0.0415474
REACTOME_REGULATION_OF_INSULIN_LIKE_GROWTH_FACTOR_IGF_TRANSPORT_AND_UPTAKE_BY_INSULIN_LIKE_GROWTH_FACTOR_BINDING_PROTEINS_IGFBPS	1.7637457	0	0.0416504
NELSON_RESPONSE_TO_ANDROGEN_UP	1.7623998	0.006437768	0.0420901
POOLA_INVASIVE_BREAST_CANCER_DN	1.7621318	0	0.0420186
HESS_TARGETS_OF_HOXA9_AND_MEIS1_DN	1.7614987	0.002096436	0.0421291
HERNANDEZ_MITOTIC_ARREST_BY_DOCETAXEL_2_DN	1.7599777	0.015904572	0.042622
REACTOME_MISCELLANEOUS_TRANSPORT_AND_BINDING_EVENTS	1.7590274	0.002227172	0.0429279
WUNDER_INFLAMMATORY_RESPONSE_AND_CHOLESTEROL_UP	1.7588319	0.002057613	0.0428057
MOOTHA_GLYCOLYSIS	1.7584457	0.01091703	0.0428286
WESTON_VEGFA_TARGETS_6HR	1.7576017	0.002202643	0.0429942
GROSS_HYPOXIA_VIA_ELK3_AND_HIF1A_UP	1.7571435	0	0.0430353
BOYLAN_MULTIPLE_MYELOMA_C_D_DN	1.7552255	0	0.0437062
MOOTHA_GLUCONEOGENESIS	1.7549627	0.004048583	0.0436351
BASAKI_YBX1_TARGETS_DN	1.7549187	0	0.0434659
OUYANG_PROSTATE_CANCER_MARKERS	1.7544974	0.008333334	0.0434524
HUMMERICH_BENIGN_SKIN_TUMOR_UP	1.7523417	0.00886918	0.0442505
REACTOME_RESPONSE_OF_EIF2AK4_GCN2_TO_AMINO_ACID_DEFICIENCY	1.75182	0	0.0443266
TURASHVILI_BREAST_LOBULAR_CARCINOMA_VS_DUCTAL_NORMAL_DN	1.7508197	0.002040816	0.044583
UZONYI_RESPONSE_TO_LEUKOTRIENE_AND_THROMBIN	1.7504139	0	0.0445738

LIU_IL13_PRIMING_MODEL	1.74849	0.014799154	0.04529
ICHIBA_GRAFT_VERSUS_HOST_DISEASE_35D_UP	1.7473887	0	0.0456067
BOWIE_RESPONSE_TO_EXTRACELLULAR_MATRIX	1.7464036	0.017738359	0.0458842
LINDVALL_IMMORTALIZED_BY_TERT_DN	1.7451417	0	0.0463767
ABE_INNER_EAR	1.7449265	0.002057613	0.0462799
REACTOME_TRANSLATION_OF_SARS_COV_2_STRUCTURAL_PROTEINS	1.7438906	0.00204499	0.0466191
STAMBOLSKY_TARGETS_OF_MUTATED_TP53_DN	1.7413279	0.002053388	0.0476478
LINDSTEDT_DENDRITIC_CELL_MATURATION_A	1.7407281	0	0.0477648
FERRANDO_TAL1_NEIGHBORS	1.7398938	0.010683761	0.0479879
CREIGHTON_ENDOCRINE_THERAPY_RESISTANCE_5	1.7396201	0	0.0479085
DIAZ_CHRONIC_MEYLOGENOUS_LEUKEMIA_DN	1.7388191	0	0.0481598
LI_INDUCED_T_TO_NATURAL_KILLER_UP	1.7368821	0	0.0488695
VERRECCHIA_RESPONSE_TO_TGFB1_C2	1.7362932	0.002114165	0.0489446
BILBAN_B_CLL_LPL_UP	1.7351054	0.006711409	0.0493579
LUI_THYROID_CANCER_CLUSTER_5	1.7343742	0.015904572	0.0494787
WP_TYPE_I_COLLAGEN_SYNTHESIS_IN_THE_CONTEXT_OF_OSTEOGENESIS_IMPERFECTA	1.7328291	0.008368201	0.0499982
REACTOME_DISEASES_OF_IMMUNE_SYSTEM	1.7328187	0.00408998	0.04981

Oncogenic signature gene sets

Gene sets	NES	NOM p-value	FDR q-value
ERBB2_UP.V1_UP	2.904524	0	0
MEK_UP.V1_UP	2.22213	0	0
LTE2_UP.V1_UP	2.185027	0	0
EGFR_UP.V1_UP	2.105998	0	0.0003639
KRAS.KIDNEY_UP.V1_DN	2.0363872	0	0.0002911
SINGH_KRAS_DEPENDENCY_SIGNATURE	1.8928977	0	0.002644
KRAS.300_UP.V1_DN	1.8229157	0	0.0052513
KRAS.600_UP.V1_DN	1.787539	0	0.0072562
LEF1_UP.V1_DN	1.7701716	0	0.0082114
KRAS.BREAST_UP.V1_UP	1.7104772	0.00203666	0.0140178

KRAS.LUNG_UP.V1_DN	1.6812701	0.002016129	0.0175686
ESC_V6.5_UP_EARLY.V1_DN	1.6506543	0.002155172	0.022199
RAF_UP.V1_UP	1.647511	0.00407332	0.0213903
ALK_DN.V1_UP	1.6474016	0.006369427	0.0199414
PKCA_DN.V1_DN	1.6291333	0.00210084	0.0224072
P53_DN.V2_UP	1.6115099	0.002040816	0.0254151
MEL18_DN.V1_DN	1.6073984	0.00210084	0.0248278
ALK_DN.V1_DN	1.5707607	0.008230452	0.0342382
KRAS.50_UP.V1_DN	1.5577035	0.02	0.0366972
EIF4E_DN	1.5452398	0.010330578	0.0392947
SNF5_DN.V1_UP	1.5420208	0.00407332	0.038521
RELA_DN.V1_DN	1.5190756	0.022403259	0.0463089
KRAS.600.LUNG.BREAST_UP.V1_DN	1.513657	0.00203252	0.0464644
STK33_UP	1.511501	0	0.0455879
AKT_UP.V1_DN	1.5032269	0.002074689	0.0469793
