# Supplementary material for: ERK MAP Kinase Signaling Regulates RAR Signaling to Confer Retinoid Resistance on Breast Cancer Cells
Source: Cancers (Basel). 2022 Nov 29;14(23):5890. doi: 10.3390/cancers14235890 (PMC9739577; doi:10.3390/cancers14235890)
Supplement: Supplementary file 1 [file cancers-14-05890-s001.zip › TableS3.pdf]

**Table S3. Gene sets significantly overlapped with RA- and ERK-upregulated genes**

**RA-upregulated genes**

| Gene set | Description | p-value | FDR q-value |
|---|---|---|---|
| HALLMARK_ESTROGEN_RESPONSE_EARLY | Genes defining early response to estrogen. | 3.37E-26 | 1.69E-24 |
| HALLMARK_ESTROGEN_RESPONSE_LATE | Genes defining late response to estrogen. | 2.13E-16 | 5.33E-15 |
| HALLMARK_UV_RESPONSE_DN | Genes down-regulated in response to ultraviolet (UV) radiation. | 1.53E-13 | 2.55E-12 |
| HALLMARK_IL2_STAT5_SIGNALING | Genes up-regulated by STAT5 in response to IL2 stimulation. | 9.72E-07 | 1.21E-05 |
| HALLMARK_TGF_BETA_SIGNALING | Genes up-regulated in response to TGFB1 [GeneID=7040]. | 1.24E-05 | 1.23E-04 |
| HALLMARK_TNFA_SIGNALING_VIA_NFKB | Genes regulated by NF-kB in response to TNF [GeneID=7124]. | 1.48E-05 | 1.23E-04 |
| HALLMARK_EPITHELIAL_MESENCHYMAL_TRANSITION | Genes defining epithelial-mesenchymal transition, as in wound healing, fibrosis and metastasis. | 1.85E-04 | 8.39E-04 |
| HALLMARK_G2M_CHECKPOINT | Genes involved in the G2/M checkpoint, as in progression through the cell division cycle. | 1.85E-04 | 8.39E-04 |
| HALLMARK_GLYCOLYSIS | Genes encoding proteins involved in glycolysis and gluconeogenesis. | 1.85E-04 | 8.39E-04 |
| HALLMARK_HEME_METABOLISM | Genes involved in metabolism of heme (a cofactor consisting of iron and porphyrin) and erythroblast differentiation. | 1.85E-04 | 8.39E-04 |
| HALLMARK_P53_PATHWAY | Genes involved in p53 pathways and networks. | 1.85E-04 | 8.39E-04 |
| HALLMARK_APOPTOSIS | Genes mediating programmed cell death (apoptosis) by activation of caspases. | 8.56E-04 | 3.57E-03 |

**ERK-upregulated genes**

| Gene set | Description | p-value | FDR q-value |
|---|---|---|---|
| HALLMARK_INTERFERON_GAMMA_RESPONSE | Genes up-regulated in response to IFNG [GeneID=3458]. | 4.89E-28 | 2.45E-26 |
| HALLMARK_INTERFERON_ALPHA_RESPONSE | Genes up-regulated in response to alpha interferon proteins. | 8.66E-26 | 2.16E-24 |
| HALLMARK_E2F_TARGETS | Genes encoding cell cycle related targets of E2F transcription factors. | 1.27E-13 | 2.12E-12 |
| HALLMARK_G2M_CHECKPOINT | Genes involved in the G2/M checkpoint, as in progression through the cell division cycle. | 7.61E-04 | 7.61E-03 |
| HALLMARK_TNFA_SIGNALING_VIA_NFKB | Genes regulated by NF-kB in response to TNF [GeneID=7124]. | 7.61E-04 | 7.61E-03 |
